# *Drosophila* Rumpelstiltskin regulates Polycomb *cis* and *trans* interactions and Homie barrier activity

**DOI:** 10.1186/s13072-026-00694-x

**Published:** 2026-08-06

**Authors:** Savanna F. Lyda, Catherine E. McManus, Juan Manuel Caravaca, Dagyeong Yang, Yuejun Wang, Hernan A. Lorenzi, Fedor Kouzine, David Levens, Leah F. Rosin, Elissa P. Lei

**Affiliations:** 1 Nuclear Organization and Gene Expression Section, Laboratory of Biochemistry and Genetics, National Institute of Diabetes and Digestive and Kidney Diseases, National Institutes of Health, Bethesda, MD USA; 2 Department of Cell Biology and Molecular Genetics, Biological Sciences Graduate Program, University of Maryland, College Park, MD, USA; 3 Program in Computational Biology, Bioinformatics, and Genomics, University of Maryland, College Park, MD USA; 4 TriLab Bioinformatics Group, National Institute of Diabetes and Digestive and Kidney Diseases, National Institutes of Health, Bethesda, MD USA; 5 Gene Regulation Section, Laboratory of Pathology, Center for Cancer Research, National Cancer Institute, National Institutes of Health, Bethesda, MD USA; 6 Present Address: Unit on Chromosome Dynamics, Division of Developmental Biology, Eunice Kennedy Shriver National Institute of Child Health and Human Development, National Institutes of Health, Bethesda, MD USA

**Keywords:** Chromatin insulator, 3D genome organization, Polycomb, Homie, PRE

## Abstract

**Background:**

Three-dimensional genome organization is maintained in part by chromatin insulators, DNA–protein complexes that create boundaries between active and inactive chromatin and regulate enhancer-promoter interactions. In *Drosophila,* the Homing insulator at *eve* (Homie) sequence prevents repressive Polycomb (PcG) chromatin at *even-skipped* (*eve*) from spreading into the neighboring essential gene, *TER94*. The exact mechanisms controlling Homie barrier activity are understudied.

**Results:**

Here, use of an in vivo reporter assay identified the requirement of the hnRNP M homolog, Rumpelstiltskin (Rump), for Homie barrier activity. Interestingly, Rump depletion resulted in no changes to chromatin insulator protein binding at Homie but extensive chromatin association changes to the Polycomb response element (PRE)-associated protein Crooked legs (Crol) at Crol-bound PREs. Furthermore, global chromatin association changes were detected for a subset of Polycomb repressive complex 1 and 2 (PRC1 and PRC2) components. Finally, we observed increased *cis-*compaction at Polycomb domains genome-wide including *eve* and increased distances between *eve* and other Polycomb domains *in trans* after Rump depletion.

**Conclusions:**

These data identify Rump as a novel regulator of Homie barrier activity and suggest a regulatory role of Rump for both *cis* and *trans* interactions of PcG proteins. Importantly, this regulatory role likely helps to maintain Homie barrier activity independently of chromatin insulator proteins.

**Supplementary Information:**

The online version contains supplementary material available at 10.1186/s13072-026-00694-x.

## Background

The interphase genome is organized in 3D within the nucleus at various scales ranging from large chromosomal territories to specific enhancer-promoter loops. Chromatin spatially segregates into either transcriptionally active or repressive compartments [1]. Furthermore, regions of the genome that are highly self-interacting are termed topologically associating domains (TADs), and the genes located within a specific TAD often display similar histone marks and transcriptional status [2]. For example, TADs marked by the repressive histone modification, H3K27me3, and the associated PcG proteins are referred to as Polycomb domains. These Polycomb domains have been shown to form long-range interactions and preferentially localize into large PcG foci in 3D space [3–6]. Polycomb domain formation occurs through the activity of two complexes, PRC1 and PRC2, neither of which contain DNA-binding domains, meaning they must be recruited to chromatin by other factors. In *Drosophila,* PcG complexes are nucleated at DNA *cis*-regulatory elements termed PREs, which contain binding domains for site-specific PRE-binding proteins [7]. Once recruited, PRC2 trimethylates H3K27, which further recruits PRC1, leading to compaction of the Polycomb domain and preferential 3D clustering [8, 9]. The compaction of Polycomb domains both restricts access to transcriptional activators and promotes H3K27me3 spreading, and both aspects are crucial for proper transcriptional repression. The spreading of H3K27me3 can be impeded either by active transcription at the domain boundary or by DNA–protein complexes termed chromatin insulators [10].

While CCCTC-binding factor (CTCF) is the primary mammalian chromatin insulator protein, *Drosophila* harbors a variety of chromatin insulator complexes. One well-studied *Drosophila* chromatin insulator is the *gypsy* insulator complex, named for its association with the *gypsy* retrotransposon, which contains 12 binding sites for the DNA-binding protein Suppressor of Hairy wing (Su(Hw)) [11]. In addition to Su(Hw), the *gypsy* complex is composed of Modifier Mdg4 isoform 67.2 [12, 13], and Centrosomal Protein 190kD (CP190), a universal component of various insulator complexes. Su(Hw) is largely enriched at intergenic regions and at the boundaries of H3K27me3 [14], supporting a model in which *gypsy* complexes function as barriers to prevent the spread of repressive chromatin. In addition to Su(Hw), binding of *Drosophila* CTCF is enriched and often aligned with CP190 and other chromatin insulator proteins near H3K27me3 boundaries [10, 15], such as within the bithorax locus [16]. Another important example of insulator binding at a Polycomb domain boundary occurs at the developmentally relevant insulator sequence*,* Homie. Homie, along with another upstream insulator site, Neighbor of Homie (NHomie), function as flanking boundaries to the Polycomb domain that spatiotemporally repress *eve*. Homie and NHomie interact in *cis* to create a stem-loop, isolating interactions between *eve* and its *cis* regulatory elements and reinforcing the *eve* TAD [17–19]. Homie forms the boundary between *eve* and the neighboring ubiquitously expressed and essential gene *TER94* [17, 20]. Homie is bound by several chromatin insulator proteins [15, 21–25], and it has been speculated that the ability to recruit many architectural proteins to a single site strengthens its activity [25]. The functional requirements of Su(Hw)- and CP190-binding at Homie have been recently explored [26], but mechanisms by which these or other unidentified *trans-*acting factors affect Homie function remain unknown. The *Drosophila* hnRNP M homolog, Rumpelstiltskin (Rump), also associates with Homie and was previously identified as a tissue-specific antagonist of the *gypsy* insulator complex [27]. Rump was originally characterized with respect to its functions in mRNA localization and splicing [28–31]. However, whether Rump plays a role in Homie barrier function is unknown.

Here, by analyzing both transgenic and endogenous Homie function, we demonstrated that Rump promotes Homie barrier activity. At endogenous Homie, chromatin association of chromatin insulator proteins is unaffected after Rump depletion. Instead, loss of Rump leads to globally altered chromatin association of the PRE-associated protein Crol, which exhibits decreased binding at the PRE upstream of Homie. We additionally observed subtle changes in the global chromatin association of PRC1 proteins and the PRC2 methyltransferase. Using the chromatin conformation capture (3C) assay, we found increased *cis*-interactions within the *eve* Polycomb domain after Rump depletion. At the same time, using Oligopaint DNA FISH we observed decreased 3D *trans-*interactions between the *eve* Polycomb domain and two other Polycomb domains. Finally, we performed Hi-C analysis to confirm the 3C and FISH results on the genome-wide level. Together, these data suggest that Rump might promote Homie barrier activity independently of chromatin insulators by affecting the formation of Polycomb domains in *cis* and 3D interactions between Polycomb domains in *trans*.

## Methods

### RNA-seq

RNA from Kc167 cells was extracted with RNeasy Mini Kit (Qiagen) according to the manufacturer’s protocol and used to generate RNA-Seq libraries. Unfragmented RNA-Seq libraries were prepared using the Ovation RNA-seq Systems 1–16 for Model Organisms (Nugen) kit from 60–100 ng of RNA, as detailed previously [32].

### RT-qPCR

Total RNA was extracted from Kc167 cells using the RNeasy Mini Kit (Qiagen). Reverse transcription of 2 μg of total RNA was performed using random primers and SuperScript III reverse transcriptase (Invitrogen) using the manufacturer’s protocol. Transcript levels were quantified by real-time PCR using HotStart-IT SYBR green qPCR Master Mix (USB Corporation). Data are represented as fold change of transcripts normalized to *RpL38*, *RpL35*, or *Tubulin*.

### Antibodies and antibody production

For Western blotting, guinea pig α-CP190 [24] was used at 1:10,000, guinea pig α-Su(Hw) [24] at 1:7,500, mouse α-Rump (Developmental Studies Hybridoma Bank 5G4) at 1:5,000, rabbit α-CTCF at 1:1,000 (generated similarly as in [33, 34], mouse α-Tubulin (Sigma T6074) at 1:100,000, mouse α-Fibrillarin (Novus 38F3) at 1:1,000, and rat α-Crol [35] at 1:2,000 dilution. All secondary HRP antibodies (Jackson ImmunoResearch) were used at 1:2,000 dilution. For ChIP-seq, the amounts of antibody used per IP were based upon recommendations from the lab of origin or manufacturer’s protocols. The sources for each antibody can be found in Additional File 9.

### ChIP-seq

2–4 × 10^7^ control or Rump depleted Kc167 cells were fixed by adding 16% PFA to the cells in culture media to final 1% formaldehyde concentration and were incubated for 10 min at RT with gentle shaking. Fixation was quenched by adding 0.125 M glycine and continuing to shake for 5 min at RT. Cells were then spun at 600 × *g* for 5 min and washed twice in cold PBS. After the second wash, chromatin was prepared using previously described methods [21] with the following modifications. Cells were incubated in cell lysis buffer for 10 min on ice before spinning and sonication using the Bioruptor (Diagenode) using 15 cycles of 30 s on and 30 s off. ChIP was completed using methods described in [21] with antibody and sepharose bead (Cytiva) specifications found in Additional File 9. For some IPs ChIP-grade Protein A/G magnetic beads (Thermo Fisher Scientific) were used; details are outlined in Additional File 9. Libraries were prepared according to the manufacturer’s protocol (NEB Ultra II for DNA Library Prep Kit). Finally, apart from H3K27me3, all samples were sequenced on Novaseq 6000 (Illumina) using 50 bp single- or paired-end sequencing at the NHLBI DNA Sequencing and Genomics Core Facility. H3K27me3 was sequenced on HiSeq2500 (Illumina) at the NIDDK Genomics Core Facility using 50 bp single-end sequencing.

### Hi-C libraries

Hi-C libraries were prepared following the optimized Hi-C 2.0 protocol described by Belaghzal et al. [36] and adapted for *Drosophila* cells. 4–5 × 10^7^ control or Rump Kc167 cells were crosslinked with 1% formaldehyde to preserve chromatin interactions, quenched with 0.125 M glycine, and washed twice with cold PBS. Cells were snap-frozen in liquid nitrogen and stored at − 80 °C until further processing.

Crosslinked cells were lysed in cold lysis buffer for 30 min, and nuclei were isolated for subsequent restriction digestion. Chromatin was permeabilized in 1 × NEBuffer 3.1 containing 0.1% SDS for 10 min at 65 °C, followed by quenching with 1% Triton X-100. DNA was digested with the 4-base cutter DpnII (NEB) to generate frequent restriction fragments for high-resolution mapping. The resulting 5′ overhangs were filled in using Klenow DNA polymerase (NEB) in the presence of biotin-14-dATP (Invitrogen) to label fragment ends.

Proximity blunt-end ligation was performed in situ using T4 DNA ligase (Invitrogen) to favor ligation of spatially proximal DNA ends. After ligation, crosslinks were reversed by incubation at 65 °C with Proteinase K (NEB), and DNA was purified by repeated phenol:chloroform extraction followed by ethanol precipitation and concentration using 30 kDa Amicon columns.

Biotin was removed from unligated ends using T4 DNA polymerase (NEB) in the presence of dATP and dGTP (Invitrogen) to enrich for valid ligation products. Free nucleotides including biotin-14-dATP were removed by repetitive concentration with Amicon columns. DNA was then sheared to ~ 200–300 bp fragments using the Bioruptor (Diagenode). Biotinylated ligation junctions were captured using Dynabeads MyOne Streptavidin C1 (Invitrogen). Sequencing libraries were prepared using the TruSeq ChIP Library Preparation Kit (Illumina), and AMPure XP beads (Beckman Coulter) selection was used to remove adapter and primer dimers.

Libraries from 2 replicates of each condition were subjected to 50 bp paired-end sequencing for genome-wide contact mapping. This optimized protocol increases the fraction of valid intra-chromosomal interaction pairs and enables high-resolution characterization of chromatin architecture.

### Bioinformatic analyses

#### RNA-seq

RNA-seq data are available at GSE308570 . FASTQ files were trimmed using Trim Galore (v0.6.10) with default settings (Illumina adapter removal, Phred quality cutoff of 20, minimum length of 20 bp, and minimum overlap of 1 bp). Trimmed reads were mapped to the FlyBase r6-52 reference genome using HISAT2 v2.2.1 with default parameters [37]. To remove rRNA-derived reads, trimmed reads were aligned to the SILVA 138 rRNA reference genome using HISAT2 v2.2.1, and rRNA-mapped reads were excluded from the primary alignments. Alignments were sorted and reads with a mapping quality below 20 were removed using samtools v1.17 [38]. PCR duplicates were removed using Picard MarkDuplicates v3.0.0 (Broad Institute). Gene expression quantification was performed using featureCounts (Subread v2.0.6) with parameters “-t exon -s 1” for this sense-stranded library [39]. Differential expression analysis was performed using DESeq2 v1.40.2 [40].

#### ChIP-seq

ChIP-seq data are available at GSE308569. FASTQ files were trimmed using Trim Galore (v0.6.7) with default settings (Illumina adapter removal, Phred quality cutoff of 20, minimum length of 20 bp, and minimum overlap of 1 bp). Trimmed reads were aligned to the FlyBase r6-52 dm6 genome assembly using Bowtie2 v2.5.3 with default settings [41]. Alignments were sorted using samtools v1.21, and reads with a mapping quality below 20 were discarded [38]. The rRNA-derived reads were identified by alignment to rRNA references, converted to BED format using bedtools [42], and excluded from the filtered BAM files with BBMap’s filterbyname.sh. PCR duplicates were removed using Picard MarkDuplicates v3.3.0 (Broad Institute). Peaks were called using MACS2 v2.2.7.1 on individual replicate BAM files of IPs and corresponding inputs [43], and replicate peaks were merged based on overlapping regions using bedtools.intersect.

### Alternative splicing

Single-end RNA-seq reads were first trimmed to remove adaptor sequences using Trimmomatic v0.39 [44] with default parameters. Clean reads were then aligned to the *D. melanogaster* reference genome (BDGP6, Ensembl release 46) using STAR v2.7.11b [45] with default settings and gene annotations from the corresponding GTF (BDGP6.46.112). Differential alternative splicing between control and Rump depleted conditions was quantified using rMATS-turbo v4.1.2 [46], specifying a read length of 50 bp and enabling multi-threading. Representative significant splicing events were visualized as sashimi plots with rmats2sashimiplot (bundled with rMATS-turbo).

### Hi-C

Hi-C data are available at GSE325266. Reads were trimmed with fastp (v0.24) and mapped to the *Drosophila melanogaster* dm6 r6.52 reference genome using the Juicer pipeline (v1.6;[47]) with default parameters. This produced one.hic contact map per sample, which was binned to generate contact matrices at resolutions ranging from 5 to 25 Kb for further downstream analyses. Hi-C contacts ranged from 258 to 664 million per matrix. Condition-level merged.hic files (Control and Rump depletion) were generated from the corresponding replicate.hic files using the Juicer Tools pre utility. Hi-C contact matrices were analyzed to investigate chromatin interactions among Polycomb domains in *Drosophila melanogaster* (dm6 assembly).

Hi-C contact matrices were extracted from Juicer.hic files using strawr (v0.0.92) at 5, 10, and 25 Kb resolution and visualized using the Juicebox Web App (v2.3.6; [48]). For intrachromosomal interactions, observed/expected (O/E) normalization was used to account for distance-dependent contact decay; for interchromosomal interactions, raw observed contact values were used. Knight–Ruiz (KR) matrix balancing was applied in all analyses. To compare chromatin contacts between conditions, difference matrices were computed by element-wise subtraction: control minus Rump depletion (Control − *rump*^*RNAi*^) using O/E values for intrachromosomal contacts and using raw observed values for interchromosomal contacts. Positive values therefore indicate stronger contacts in the control condition.

Genomic regions with low sequencing coverage or potential technical artifacts were excluded using two criteria: (i) known blacklisted regions in the dm6 assembly [49] were removed; and (ii) low-contact bins with fewer than 2 raw contacts (summed across all partners) were excluded to reduce noise from poorly covered regions. For differential analyses, bins not meeting the contact threshold in at least one condition were discarded.

All Polycomb domains identified by H3K27me3 ChIP-seq were analyzed across chromosome arms 2L, 2R, 3L, 3R, and X. The null distribution compared mean contacts between each Polycomb domain pair to contacts between each Polycomb domain and randomly sampled size-matched genomic intervals from the same chromosome arm as the second Polycomb domain, excluding blacklisted and low-contact bins.

Contact enrichment within individual Polycomb domains was assessed by comparing the distribution of O/E values among bin pairs within each Polycomb domain to bin-pair O/E values within randomly sampled, size-matched non-Polycomb genomic regions (excluding blacklisted and low-contact bins).

### Oligopaint DNA FISH

Oligo pools were purchased from Twist biosciences (San Francisco, CA), and Oligopaints were synthesized by PCR-based amplification of the barcodes on each oligo as previously described [50]. Oligopaints for *Antp*, *Abd-B*, Ctrl A and Ctrl B were designed to have 42 bp of homology with a probe density of ~ 10 probes per Kb. The Homie Oligopaint was designed to have 60 bp of homology and a probe density of ~ 7 probes per Kb. Additional details regarding genomic locations and probe sizes can be found in Additional File 8.

Control and Rump depleted Kc167 cells were applied in parallel onto the same poly-L-lysine-treated slide and fixed with 4% paraformaldehyde in PBST (PBS with 0.1% Triton X) for 15 min at RT. Slides were washed 3 × in PBST for 5 min per wash. Slides were next permeabilized in PBS with 0.5% Triton X for 15 min then washed in 2X SSCT for 5 min. Slides were then incubated in 50% formamide in 2X SSCT for 5 min at RT, for 2.5 min at 92 °C, and for 20 min at 60 °C. Slides were then allowed to dry and cool for 10 min before adding hybridization mix. The hybridization mix was composed of 50–100 pmol of each probe being tested, along with 1.5 μL of 25 mM dNTPs. The probes and dNTPs were dried and resuspended in 12.5 μL of 100% formamide, 5 μL of PVSA, 1μL of RNase A, and 6.5 μL of DNA hybridization buffer to a final volume of 25 μL. This hybridization mix was then added to each slide and mounted with a coverslip before being denatured on a 92 °C heat block for 2.5 min, then hybridized overnight at 37 °C in a humidified chamber. The next day, slides were washed in 2X SSCT for 15 min at 60 °C and for 15 min at RT, followed by one wash in 0.2X SSC for 5 min at RT. 10 pmol of each fluorophore-conjugated secondary probe was hybridized for 2–3 h at 37 °C in a humidified chamber. Slides were washed after the primary probe incubation, followed by a 5 min wash of 1:10,000 DAPI in PBS and two final 5 min washes in PBST. Slides were mounted with Prolong Diamond (Life Technologies) and sealed with coverslips.

Images were captured on a Leica DMi 6000B widefield fluorescence microscope using a 63 × oil-immersion objective and a Leica DFC9000 sCMOS Monochrome Camera with DAPI, GFP, CY3, and CY5 filter cubes. All images were captured at a 0.24 μm XY resolution and a 0.426 μm Z resolution with final images of 2048 × 2048 pixels. Exposure times varied slightly per experiment based upon probe signal but were kept consistent across conditional replicates. The general exposure times are as follows: PcG and control probes after Rump depletion (DAPI: 25 ms, GFP: 200 ms, CY3: 100-150 ms, CY5: 200 ms), PcG and control probes after CTCF depletion (DAPI: 20-35 ms, GFP: 250-350 ms, CY3: 100-150 ms, CY5: 250-300 ms), and PcG and control probes after CP190 depletion (DAPI: 30 ms, GFP: 300 ms, CY3: 150 ms, CY5: 250-300 ms). After deconvolution using Huygens Professional software (Scientific Volume Imaging), images were segmented and measured by the TANGO 3D-segmentation plugin for ImageJ (NIH). Only cells with one focus corresponding to each probe were analyzed. Additionally, we analyzed > 1000 cells from each replicate with a total of 3 replicates.

### *Drosophila* strains

*Drosophila* stocks were maintained at 25 °C on standard cornmeal medium. *Act5C-*Gal4, *da-*Gal4, *Mef2-*Gal4, and *l(3)31-1-*Gal4 driver lines as well as w^1118^; DCTN1-p150^1^/TM3, P{w[+ mC] = sChFP}3, Sb^1^ (#35524) were obtained from the Bloomington *Drosophila* Stock Center. The *rump*^*1*^ line [29] was a gift from E. Gavis. Lines expressing dsRNA against *rump* (44658 GD)*, Cp190* (35078 GD)*, ctcf* (30713 GD) and *su(Hw)* (10724 GD) were obtained from the Vienna *Drosophila* RNAi center. The *TER94-GFP* lines [20] were a gift from J. Jaynes. Protein extracts from 3rd instar larval anterior thirds were used for Western blotting, except for *Cp190* RNAi, for which whole 1st and 2nd instar larvae were used.

### Embryo collections

Embryos aged 22–24 h were collected from 2 h egg lays on molasses plates. The *rump*^*1*^ mutation was balanced with TM3, P{w[+ mC] = sChFP}3, Sb^1^, which expresses mCherry ubiquitously under control of the *sph* promoter. Homozygous mutant (RFP-) embryos were selected using RFP fluorescence. Embryos were dechorionated and frozen on dry ice before RNA was extracted using Trizol (Thermo Fisher Scientific).

### Cloning of the Homie insulator

The 369 bp region corresponding to the Homie insulator (chr2R:9,988,750–9,989,118; [51]) was amplified from OregonR genomic DNA and cloned sequentially into the pLucky *attB* vector (gift from M. Markstein) to flank *UAS::phsp70::Luciferase* at *NheI* and *SpeI* sites. The resulting Homie-insulated constructs were integrated at the *attP3* landing site, which is shown to harbor repressive chromatin in both the Kc167 cell line and *Drosophila* embryos ([5, 6], Additional Fig. 3) by *phiC31* integrase by BestGene.

### Luciferase barrier assay

Luciferase insulator barrier activity assays were performed as described [24, 27]. Male 3rd instar larvae were frozen on dry ice and stored at -80 °C until use. Individual larvae were ground using an electric pestle in 30 (*Mef2* and *l(3)31–1* drivers) or 50 µL (*Act5C* and *da* drivers) of Glo Lysis Buffer (Promega) for 30 s and incubated at RT for 10 min. Samples were then spun at max speed for 5 min, and 15 µL were loaded onto an opaque 96-well plate. 15 µL of Bright Glo reagent (Promega) was added to each well and luciferase activity was detected using a Spectramax II Gemini EM plate reader (Molecular Devices). Luciferase activity was normalized to total protein for each well as determined by BCA protein assay (Thermo Scientific).

### Cell lines

Kc167 cells were grown in CCM3 media (HyClone) and maintained in monolayer at 25 °C. 1 × 10^7^ cells were transfected with 2 µg of either GFP (control), *rump, Cp190,* or *CTCF* dsRNA with the Nucleofector Kit V (Lonza) using program G-30.

### Chromatin conformation capture

4 × 10^6^ control or Rump depleted Kc167 cells were crosslinked in 1% formaldehyde in CCM3 media for 10 min at RT and quenched with 0.125 M glycine for 5 min at RT. Samples were incubated on ice for 5 min and then spun at 1,200 x*g* at 4 °C for 5 min. Cells were washed with 5 mL of cold PBS and spun again. Lysis was performed by incubating cells in 1 mL of lysis buffer [10 mM NaCl, 0.2% NP-40, 10 mM Tris pH 8 with cOmplete protease inhibitor cocktail (Roche)] at 37 °C for 20 min. Samples were spun at 1,200 x*g* at 4 °C for 5 min, and the lysis step was repeated with another 1 mL of lysis buffer. Pellets were then washed with 1 mL of digestion buffer [0.2% NP-40, 1X CutSmart Buffer (NEB)] and spun at 1,200 × g at 4 °C for 5 min. Nuclei were resuspended in 1.6 mL of digestion buffer supplemented with 0.1% SDS and incubated at 65 °C for 30 min. Triton X-100 was added to a final concentration of 1%, and samples were incubated at 37 °C for 15 min. Samples were digested with 1600 U of EcoRI-HF (NEB) at 37 °C overnight. The next day, samples were incubated for 20 min at 65 °C to inactivate *EcoRI-HF*. Samples were then diluted to 4 mL with ligation buffer [1% Triton X-100, 1X T4 DNA Ligase Reaction Buffer (NEB)] and incubated at 37 °C for 30 min. 4800 U of T4 DNA Ligase (NEB) was added to each sample, followed by incubation at 16 °C overnight. The next day, proteinase K was added to a final concentration of 65 µg/mL to all samples. Samples were then incubated at 65 °C overnight to reverse crosslinking. The next day, 1 volume of phenol:chloroform:isoamyl alcohol (25:24:1) was added to each sample. The samples were vortexed for 15 s and centrifuged for 10 min at 4400 rpm. The aqueous layer was transferred to a new tube, and 1 volume of chloroform was added. The samples were vortexed and spun again, and the aqueous layer was transferred to a new tube. 1 volume of water, 0.1 volume of 3 M NaOAc pH 5.2, 2.5 volumes of 100% ethanol, and 2 μL of Glycoblue (Ambion) was added to each sample. After incubating for 1 h at -80 °C, samples were spun for 20 min at 4 °C at 10,000 x*g*. Pellets were washed with 75% ethanol and spun again for 5 min at 4 °C at 4,400 rpm. Pellets were air dried at RT prior to resuspension in 350 μL TE. Concentrations of samples were normalized by SYBR Green qPCR to the *drl* locus. Interaction frequencies were detected by TaqMan qPCR using a TaqMan MGB probe located ~ 1.6 Kb upstream of Homie. TaqMan Ct values were normalized to standard curves generated using a digested and re-ligated BAC covering the analyzed region (CH321-01G04, BACPAC).

## Results

### Rump regulates *TER94* expression through the Homie insulator

**Fig. 1:**
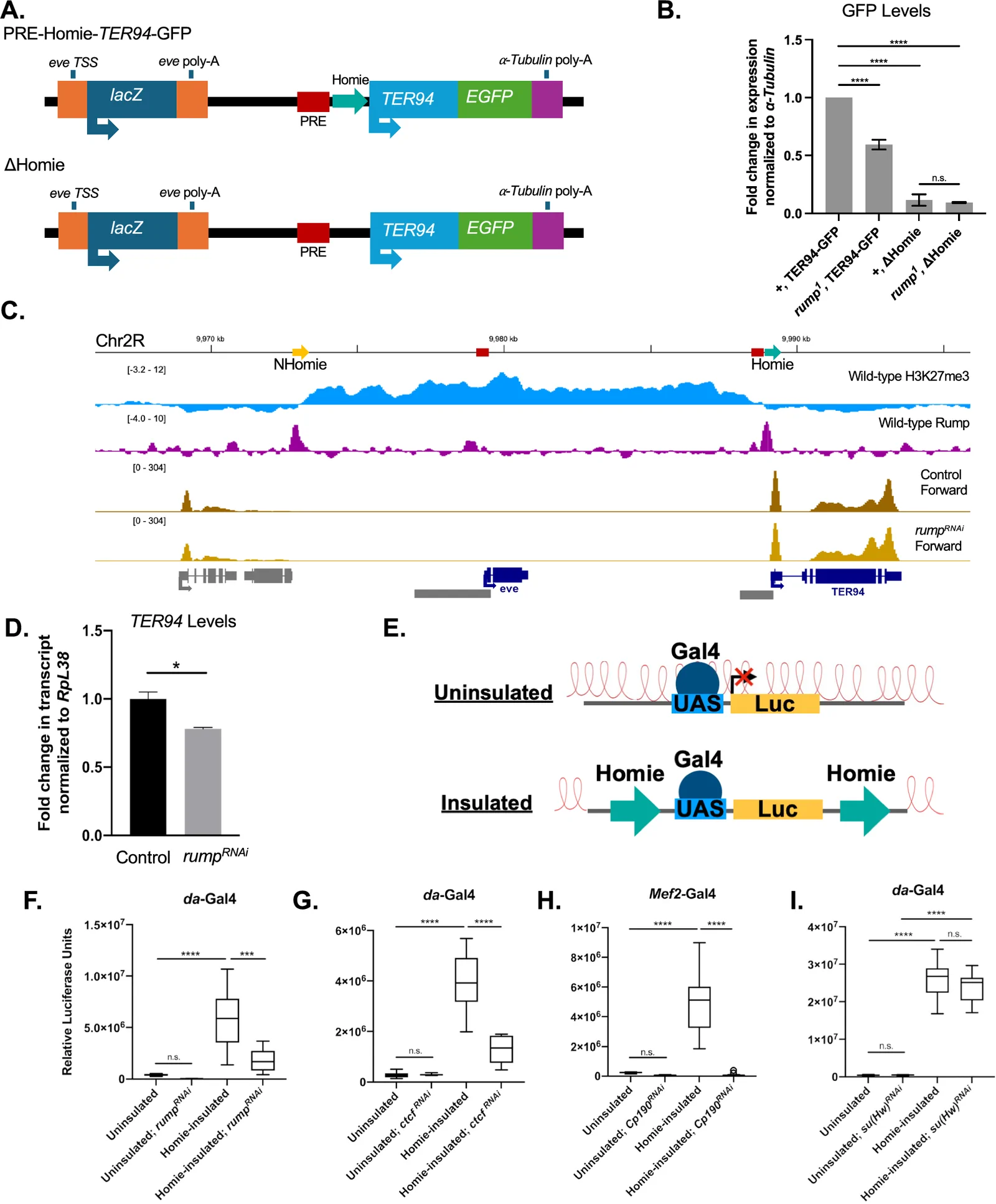
Rump regulates *TER94* expression; and Rump, CTCF, and CP190 promote Homie barrier activity. **A** Schematic showing the PRE-Homie-*TER94-GFP* transgene with and without the Homie sequence (ΔHomie). **B** RT-qPCR for *GFP* mRNA expression in wildtype or rump^1^ null embryos expressing a *TER94-GFP* transgene normalized to the housekeeping gene, *α-Tubulin*. Bar graph represents the mean of *GFP* expression with standard deviation represented by error bars (n = 3 biological replicates; **** *p* < 0.0001, n.s, not significant p-value > 0.05, Student’s t-test). **C** H3K27me3 and Rump ChIP-seq from control Kc167 cells and RNA-seq tracks from Kc167 cells in control (top) and *rump*^*RNAi*^ (bottom) conditions at the *eve-TER94* locus. NHomie and Homie are marked by yellow and teal arrows, respectively, with orientation indicated relative to their endogenous sequence orientation. PRE sites are marked by red boxes. *TER94* is downregulated after Rump depletion (log_2_FC: − 0.21, p_adj_: 0.040). **D** RT-qPCR for *TER94* mRNA expression in control and *rump*^*RNAi*^ Kc167 cells normalized to the housekeeping gene, *RpL38*. Bar graph represents the mean of *TER94* expression with standard deviation represented by error bars (n = 4 biological replicates; *, *p*-value < 0.05, Student’s t-test). **E** Schematic of luciferase reporter assay. UAS-*Luciferase* transgenes are uninsulated or insulated (flanked by the Homie sequence in its endogenous (forward) orientation). **F**–**I** Box-and-whisker plot of luciferase barrier assay using the uninsulated or Homie-insulated line in both control and *rump*^*RNAi*^ (**F**), *CTCF*^*RNAi*^ (**G**), *Cp190*^*RNAi*^ (**H**), or *su(Hw)*^*RNAi*^ (**I**) conditions. The UAS-GAL4 system is driven by the ubiquitous *daughterless* (*da*) promoter for *rump*^*RNAi*^, *CTCF*^*RNAi*^, and *su(Hw)*^*RNAi*^ while *Cp190*^*RNAi*^ uses the *Mef2* promoter. Relative luciferase is normalized to total protein levels (n = 12 male larvae per genotype; ****, *p* < 0.0001, **, *p* < 0.005, n.s, not significant *p*-value > 0.05, one-way ANOVA followed by Tukey post hoc test). The median is indicated by a line between the lower and upper quartiles, while the whiskers represent the min and max of each data set.

Given the known functional relationship between Rump and the *gypsy* insulator complex, and the chromatin association of Rump and *gypsy* insulator proteins with the Homie insulator (Additional Fig. 1), we hypothesized that Rump has a role in regulating Homie insulator function. To test the effect of Rump on Homie barrier activity, we used a previously described transgene integrated on chr3R that contains the PRE-Homie-*TER94* region with GFP-tagged *TER94* serving as a reporter of Homie barrier function ([20]; Fig. 1A). We measured GFP expression from this transgene in *rump*^*1*^ null mutant embryos ([29]; Fig. 1B and Additional Fig. 2A) and confirmed loss of *rump* transcript in *rump*^*1*^ mutants by RT-qPCR (Additional Fig. 2B). GFP levels relative to *α-Tubulin* are significantly decreased in *rump*^*1*^ mutants compared to wildtype, confirming that Rump promotes *TER94* expression in this transgenic context (Fig. 1B). We confirmed this decrease in GFP levels by alternatively normalizing to a second housekeeping gene, *RpL35* (Additional Fig. 2A). We also examined GFP levels for a second transgene inserted in the same position termed *ΔHomie*, which is identical except that Homie is deleted ([20]; Fig. 1A). In line with previous data, we observe loss of barrier activity and dramatic reduction of GFP mRNA levels for *ΔHomie* compared to the original PRE-Homie-*TER94-GFP* transgene ([20]; Fig. 1B and Additional Fig. 2A). Furthermore, we observed no significant difference in levels of GFP expression for the *ΔHomie* transgene when comparing *rump*^*1*^ to control, consistent with Rump promotion of *TER94* expression being dependent on the presence of Homie (Fig. 1B and Additional Fig. 2A). Importantly, RNA-seq analysis after dsRNA-mediated depletion of Rump protein in embryonic derived Kc167 hematocyte cells (Kc167 cells) identified *TER94* as one of 452 significantly downregulated genes (log_2_FC: − 0.21, p_adj_: 0.040; Fig. 1C, Additional Fig. 2c, Additional File 2-3). This reduction in endogenous *TER94* expression after Rump depletion was confirmed by directed qRT-PCR (Fig. 1D). Importantly, RNA splicing analysis shows that *TER94* does not undergo any significant alterations in splicing upon Rump depletion (Additional File 6). Together, these results suggest that Rump might promote *TER94* expression in both transgenic and endogenous contexts through regulation of Homie barrier activity.

### Rump, CP190, and CTCF are required for Homie barrier activity

To test directly the role of Rump in Homie barrier activity, we designed a quantitative in vivo reporter assay to easily test potential *trans-*acting factors. We designed a transgene similar to a previously established reporter assay for *gypsy* insulator barrier activity [24]. Here, our transgene contains two copies of the Homie sequence flanking an upstream activator sequence (UAS)-driven luciferase reporter (Homie-insulated) that can be activated by a GAL4 transcription factor. The reporter transgene is integrated on the X chromosome at a site harboring repressive chromatin (Additional Fig. 3); thus, if the Homie barrier in our construct is functional, this repressive chromatin will be blocked from spreading onto the luciferase reporter. However, if the Homie barrier is not functional the luciferase reporter will be repressed (Fig. 1E, Additional Fig. 4A; see Methods). We confirmed that the Homie sequence can act as a barrier in this assay by activating the reporter with a ubiquitous (*Act5C*), muscle (*Mef2*), or central nervous system (*l(3)31-1*)-enriched GAL4 driver in third-instar larvae (Additional Fig. 4B, Additional File 4). We found that luciferase expression was significantly higher when the transgene was flanked by Homie sites compared to the uninsulated control. We also observed that the Homie sequence acted as stronger barrier to repressive chromatin spreading than the *gypsy* insulated line using both *Act5C* and *I(3)31-1* GAL4 drivers. Additionally, we confirmed orientation specificity by flipping the orientation of the Homie sequence inserted upstream of the luciferase promoter (Homie-rev). Interestingly, the Homie-rev line created the least effective barrier in all tissues tested, consistent with previous results showing Homie pairing interactions to be orientation dependent [18, 26]. Therefore, we decided to use only the Homie insulated line containing two forward-oriented Homie sequences for remaining experiments.

We then performed this barrier assay in combination with UAS-driven RNAi hairpin-mediated depletion of potential *trans-*acting factors. In addition to Rump, we tested three different insulator proteins, Centrosomal Protein 190 kDa (CP190), CCCTC-binding factor (CTCF), and Suppressor of Hairy-wing (Su(Hw)). Depletion of candidate factors in larvae was verified by western blotting (Additional Fig. 4C). When depleting Rump using the ubiquitous *daughterless* (*da*) GAL4 driver, we found that luciferase expression of the Homie-insulated line is significantly decreased compared to control, supporting our earlier finding that Rump promotes Homie barrier activity (Fig. 1F). Although we also observe a small decrease of already low luciferase expression after Rump depletion in the uninsulated line, the effect is not statistically significant. In addition to Rump, depletion of CTCF using the *da-*Gal4 driver results in significant decrease in luciferase expression for the Homie-flanked transgene (Fig. 1G). Depletion of CP190 with *da-*GAL4 resulted in lethality; therefore, we used *Mef2*-GAL4, a muscle-specific driver, resulting in dramatic loss of Homie-insulated luciferase expression compared to the control (Fig. 1H). In contrast, depletion of Su(Hw) using the *da-*GAL4 driver has no significant effect on luciferase activity (Fig. 1I). These data support that Rump, CTCF, and CP190 promote Homie barrier activity.

### Genome-wide chromatin association of insulator proteins is largely unaffected after Rump depletion

**Fig. 2:**
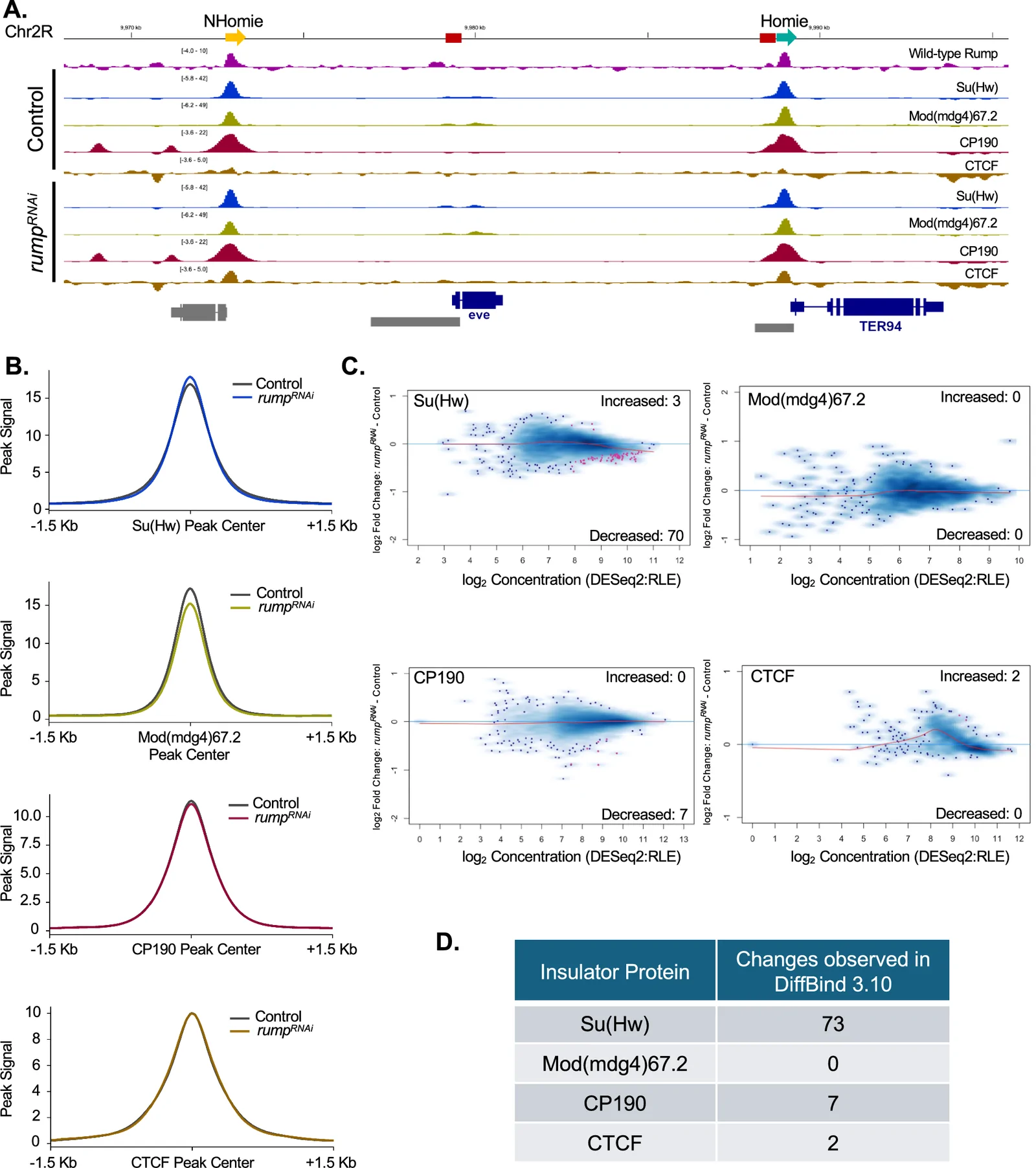
Genome-wide chromatin association of insulator proteins in Kc167 cells after Rump depletion. **A** Screenshot of Rump ChIP-seq in control cells with the *gypsy* core complex (Su(Hw), Mod(mdg4)67.2, and CP190) and CTCF (n = 3 biological replicates) merged ChIP-seq signal at the *eve-TER94* locus in Kc167 cells in both control (top) and *rump*^*RNAi*^ (bottom) conditions. **B** Average of Su(Hw), Mod(mdg4)67.2, CP190, or CTCF ChIP-seq signal plotted ± 1.5 Kb of their respective peak centers in control conditions. **C** MA-plot of differential Su(Hw) (top left), Mod(mdg4)67.2 (top right), CP190 (bottom left), or CTCF (bottom right) ChIP-seq peaks after Rump depletion detected by DiffBind 3.10. Plotted on the y-axis is log_2_FC (*rump*^*RNAi*^*-*Control) and plotted on the x-axis is log_2_Concentration using DESeq2:RLE normalization. Differential peaks (represented by pink dots) are identified using a p_adj_ cutoff of 0.05. **D** Table showing the number of differential peaks (in either direction) identified for each insulator protein after Rump depletion.

Since the effect of Rump depletion on Homie barrier activity is similar to CTCF and CP190 depletion, we tested whether Rump regulates insulator protein recruitment to chromatin. It was previously shown in wildtype Kc167 cells that 86% of Rump ChIP-seq peaks overlap with the *gypsy* insulator complex*,* including at Homie and NHomie; additionally, 50% of Rump ChIP-seq peaks overlap with CTCF binding sites [27]. Therefore, we hypothesized that binding of Rump with insulator proteins might be inter-dependent and that a loss of Rump would lead to altered chromatin association of the *gypsy* core complex or CTCF. To test this possibility, we performed ChIP-seq of each member of the *gypsy* core complex (Su(Hw), CP190, and Mod(mdg4)67.2) as well as CTCF, after Rump depletion in Kc167 cells. We examined localization specifically at the *eve* Polycomb domain as well as average signal at called peaks. Overall, Rump depletion results in no apparent change in chromatin association of the *gypsy* core complex at Homie, NHomie, or genome-wide (Fig. 2A, B). Additionally, chromatin association of CTCF genome-wide is unchanged after Rump depletion. Although CTCF signal at Homie and NHomie appears increased upon Rump depletion, its binding at these sites is extremely low relative to much stronger CTCF binding sites across the genome. In fact, CTCF binding at Homie and NHomie are not called as peaks in our analysis (Fig. 2A). Additionally, we performed DiffBind 3.10 analysis to identify individual peaks with statistically significant changes in peak strength after Rump depletion across 3 biological replicates. These analyses revealed few to no chromatin association changes for each insulator protein tested (Fig. 2C, D; Additional File 5).

### Depletion of Rump strongly affects chromatin association of the PcG recruiter, Crol

**Fig. 3:**
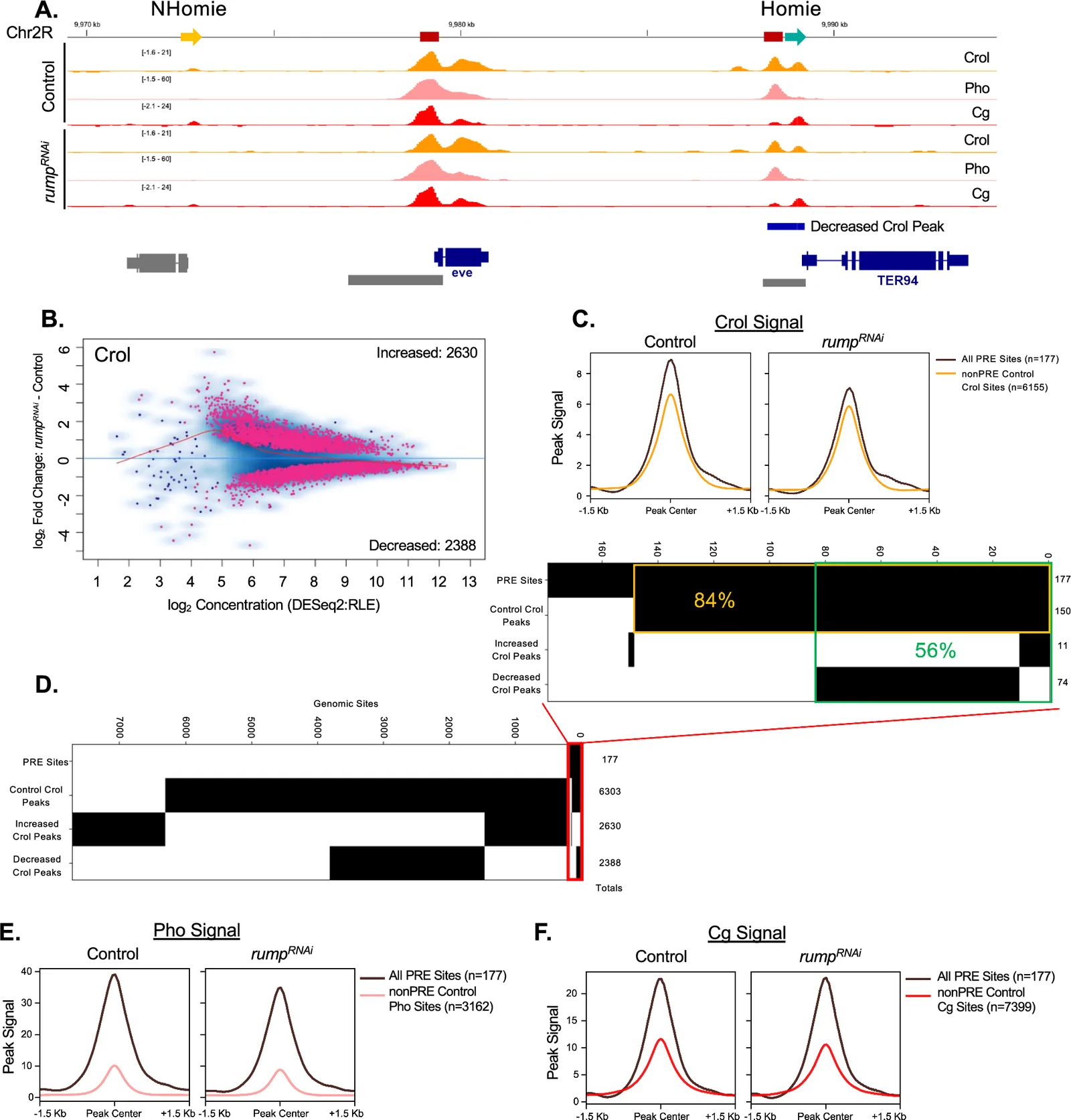
Depletion of Rump specifically alters chromatin association of the PRE-associated protein Crol. **A** Screenshot of the merged ChIP-seq signal of three PRE-binding proteins (Crol (orange), Pho (pink), and Cg (red)**;** n = 3 biological replicates), respectively, at the *eve-TER94* locus in both control (top three tracks) and *rump*^*RNAi*^ (bottom three tracks) conditions in Kc167 cells. PRE sites are marked by red boxes. Note that Crol binding at Homie and the PRE is juxtaposed and called as a single peak in our analysis. The blue box below the tracks represents a Crol peak that is decreased after Rump depletion (n = 3 biological replicates; log_2_FC: − 0.49, p_adj_: 1.7e-04). **B** MA-plot of statistically significant Crol peaks after Rump depletion. Plotted on the y-axis is log_2_FC (*rump*^*RNAi*^*-*Control) and plotted on the x-axis is log_2_Concentration using DESeq2:RLE normalization. We observed 2630 increased peaks and 2388 decreased peaks (represented by pink dots) using a p_adj_ cutoff of 0.05. **C** Average Crol ChIP-seq signal plotted ± 1.5 Kb of peak center at either all PRE sites (brown) or nonPRE control Crol sites (orange) separated by control (left) or Rump depleted (right) conditions. **D** Binary heat map of PRE sites, Crol peaks in control conditions, Crol peaks increased after *rump*^*RNAi*^, and Crol peaks decreased after *rump*^*RNAi*^. Each column represents a single genomic location, and the presence of plotted factor (row) is marked with a black line. The red box indicates only PRE regions, which are expanded for better visualization. The yellow box indicates Crol-bound PRE sites, identified by overlap of Control Crol peaks with PRE sites. The green box indicates Crol-bound PRE sites that show either increased or decreased Crol binding after Rump depletion. **E** Average Pho ChIP-seq signal plotted ± 1.5 Kb of peak center at either all PRE sites (brown) or nonPRE control Pho sites (pink) separated by control (left) or Rump depleted (right) conditions. **F** Average Cg ChIP-seq signal plotted ± 1.5 Kb of peak center at either all PRE sites (brown) or nonPRE control Cg sites (red) separated by control (left) or Rump depleted (right) conditions.

Given that Rump depletion weakens Homie barrier activity without altering chromatin association of insulator proteins, we considered that Rump might instead affect formation of the Polycomb domain upstream of Homie. Since Rump is an RNA-binding protein, we first considered the possibility of Rump-mediated indirect effects on mRNA expression or processing. To this end, we revisited the RNA-seq data after Rump depletion with respect to genes related to PcG function. Indeed, we found that the PcG recruiter gene *crol* was mildly (log_2_FC: − 0.32, p_adj_: 0.0031) but significantly downregulated according to RNA-seq after Rump depletion (Additional Fig. 5A, Additional File 3). Further analysis of the RNA-seq data showed a change in alternative splicing of the 5’ UTR of *crol* (Additional Fig. 5B, Additional File 6); however, western blotting detected no change in steady state Crol protein levels. This result suggests that the *crol* open reading frame, and translation to Crol protein, is likely unaffected at least at steady state (Additional Fig. 5C). In addition to altering *crol* RNA, we examined whether Rump might impact chromatin association of the Crol protein. To test this possibility, we performed ChIP-seq of Crol after Rump depletion and found that 80% of 6303 Crol peaks are significantly affected (FDR < 0.05), with 2630 increased and 2388 decreased peaks (Fig. 3A, D, Additional Fig. 6, and Additional File 5). Analysis of genome-wide Crol binding sites confirmed previously published data showing that while the majority of Crol binding is located outside of PREs, 84% of PREs harbor Crol binding, and Crol signal is stronger at PREs compared to non-PREs (Fig. 3C, D and Additional Fig. 6B–D; [52]). Focusing specifically on PREs, we found that 7% of Crol-bound PREs (11/150) have increased Crol binding, and 49% of Crol-bound PREs (74/150) have decreased Crol binding after Rump depletion (Fig. 3D and Additional Fig. 6D). At the *eve* Polycomb domain, we found that Crol binding is unchanged at the PRE near the *eve* promoter but decreased at the canonical PRE upstream of Homie (Fig. 3A, log_2_FC: − 0.49, p_adj_: 1.7e-04). While we also observe binding of Crol at Homie, this binding is slightly weaker than at the neighboring PRE.

Additionally, we analyzed the effects on the RNA and chromatin association of two other DNA-associated PcG recruiter proteins, Pleiohomeotic (Pho), which is important for PRE function at Homie [53], and Combgap (Cg) [54]. RNA analyses after Rump depletion revealed that overall *pho* levels were unchanged (Additional File 3, log_2_FC: − 0.033, p_adj_: 0.87) while splicing of the *pho* RNA 5’UTR was significantly altered (Additional File 6 and Additional Fig. 7). Moreover, RNA analyses of *cg* after Rump depletion revealed the opposite showing no significant splicing alterations (Additional File 6) while overall *cg* RNA was slightly downregulated (Additional File 3 and Additional Fig. 7, log_2_FC: − 0.20, p_adj_: 0.044). Nevertheless, after Rump depletion, Pho binding was only mildly decreased and not affected at the *eve* Polycomb domain, and Cg binding was unaffected overall (Fig. 3A, E, F). Few to no statistically significant changed peaks were observed for either factor genome-wide (0 Pho and 6 Cg sites, respectively, Additional Fig. 8). However, when analyzing Pho and Cg signal at differential Crol binding sites we observed that outside of PREs both Pho and Cg were increased at upregulated Crol sites and decreased at downregulated Crol sites after Rump depletion (Additional Fig. 9). These data align with previous studies highlighting a role for Crol in recruitment of PcG proteins outside of the PRE context [52]. Taken together, these data show that depletion of Rump uniquely alters the recruitment of PcG recruiter Crol and supports the established concept of functional relationships between PRE-associated proteins.

### Examination of genome-wide PcG chromatin association after Rump depletion

**Fig. 4:**
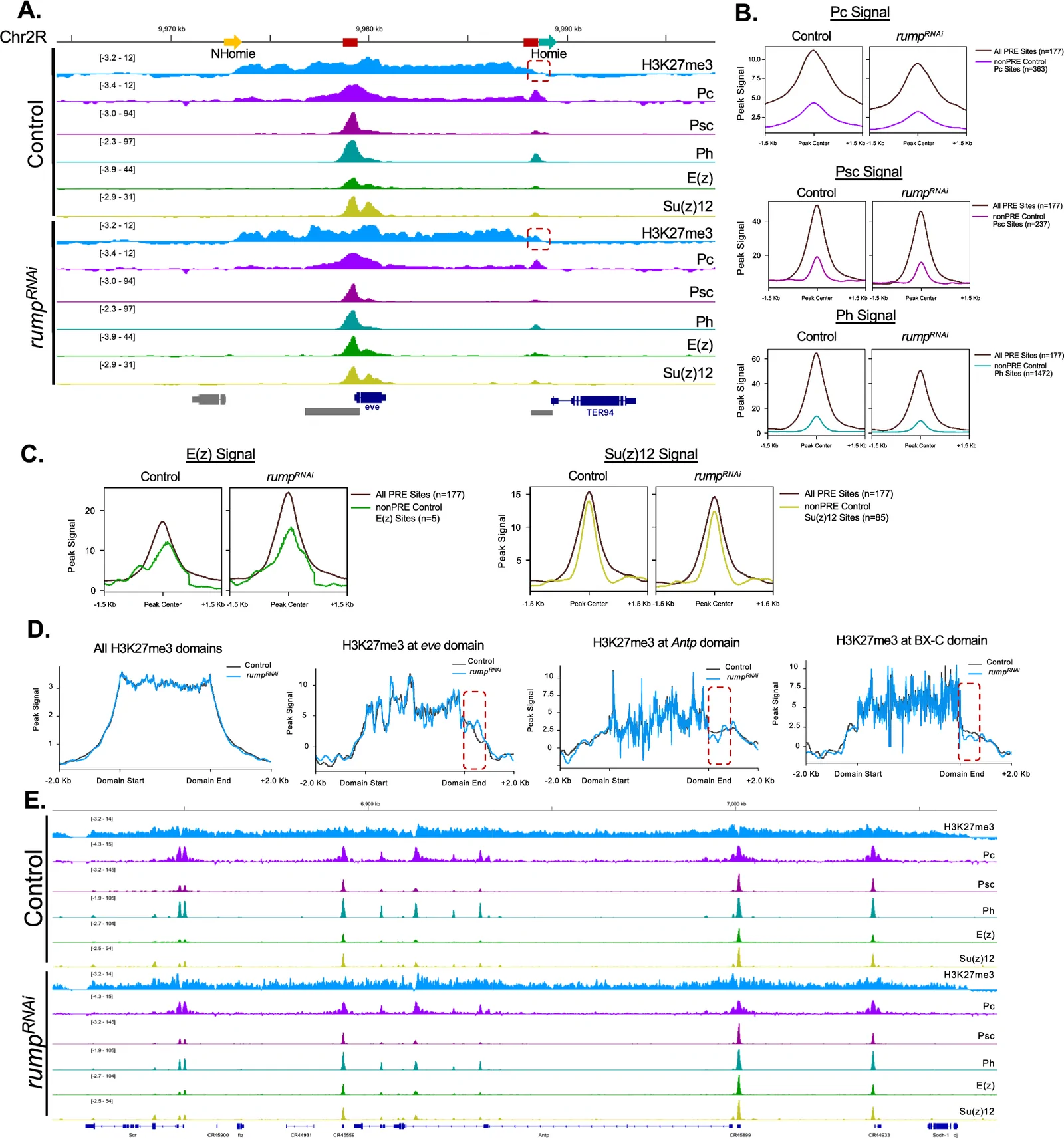
Depletion of Rump alters chromatin association of PRC1 proteins and the PRC2 methyltransferase, E(z). **A** Screenshot of H3K27me3 (n = 2 biological replicates), PRC1 protein (Pc, Psc, or Ph**;** n = 3 biological replicates), and PRC2 protein (E(z) or Su(z)12; n = 3 biological replicates) merged ChIP-seq signal at the *eve-TER94* locus in Kc167 cells in both control (top six tracks) and *rump*^*RNAi*^ (bottom six tracks) conditions. A red dashed box is drawn to aid comparison of H3K27me3 spreading between control and *rump*^*RNAi*^ conditions. **B** Average of PRC1 protein (Pc (top), Psc (middle), or Ph (bottom)) ChIP-seq signal plotted ± 1.5 Kb of peak center at either all PRE sites (brown) or nonPRE bound Pc (light purple), Psc (dark purple), and Ph (teal) separated by control (left) or Rump depleted (right) conditions. **C** Distribution of PRC2 protein (E(z) (left) or Su(z)12 (right)) ChIP-seq signal plotted ± 1.5 Kb of peak center at either all PRE sites (brown) or nonPRE bound E(z) (green) and Su(z)12 (yellow) separated by control (left) or Rump depleted (right) conditions. **D** H3K27me3 (Global average (all domains), at the *eve, Antp*, or BX-C domains) ChIP-seq signal plotted ± 2.0 Kb of H3K27me3 domain peak boundaries in control conditions. Note that H3K27me3 domains harbor a range of sizes (from 2019 to 399,483 bp), so the x-axis displayed for the global signal is not a consistent length. **E** Screenshot of H3K27me3, PRC1 protein (Pc, Psc, or Ph), and PRC2 protein (E(z) or Su(z)12) ChIP-seq signal at the *Antp* locus in Kc167 cells in both control (top six tracks) and *rump*^*RNAi*^ (bottom six tracks) conditions.

Given extensive chromatin association changes of Crol at PREs, we next analyzed chromatin association of several components of PRC1 and PRC2. First, we performed ChIP-seq of three PRC1 proteins, Polycomb (Pc), Posterior sex combs (Psc), and Polyhomeotic (Ph), after Rump depletion. When analyzing signal at all PRE sites versus nonPRE sites as well as signal at the *eve* Polycomb domain, we found chromatin association of all PRC1 proteins to be reduced particularly at PREs after Rump depletion (Fig. 4A, B, E). Next, we performed ChIP-seq of PRC2 components Enhancer of zeste (E(z)), the primary H3K27 methyltransferase, and Suppressor of zeste 12 (Su(z)12). While chromatin association of Su(z)12 appeared relatively unaffected, we observed an increase of E(z) chromatin association both genome-wide and at *eve* PRE sites (Fig. 4A, C, E). We also analyzed signal of all PRC1 and PRC2 proteins at differential Crol binding sites (Additional Fig. 9). Here we observed that Pc and E(z) signal at nonPRE Crol sites was extremely low compared to PRE sites. Alternatively, Psc, Ph, and Su(z)12 showed a mild enrichment specifically at nonPRE Crol sites that were upregulated after Rump depletion (Additional Fig. 9). Importantly, the enrichment for Psc, Ph, and Su(z)12 signal itself was altered between control and Rump depleted conditions. Furthermore, similar to insulator proteins, very few significantly differential peaks were detected for PRC1 and 2 proteins (Additional Fig. 8). Finally, given the mild increase of E(z) genome-wide, we hypothesized that there might also be an increase of the repressive histone mark H3K27me3 after Rump depletion. Indeed, ChIP-seq of H3K27me3 after Rump depletion shows slight spreading of H3K27me3 from the *eve* Polycomb domain onto *TER94*; however, spreading is not observed when analyzing Polycomb domains genome-wide in meta-analyses or for other specific Polycomb domains (Figs. 4A, D, E, and Additional Fig. 10). These data are consistent with the possibility that Rump functions to maintain *eve* Polycomb domain formation and promote Homie barrier activity, preventing the spread of repressive H3K27me3 onto *TER94*.

### Depletion of Rump but not insulator proteins results in increased *cis*-compaction of the *eve* polycomb domain

**Fig. 5:**
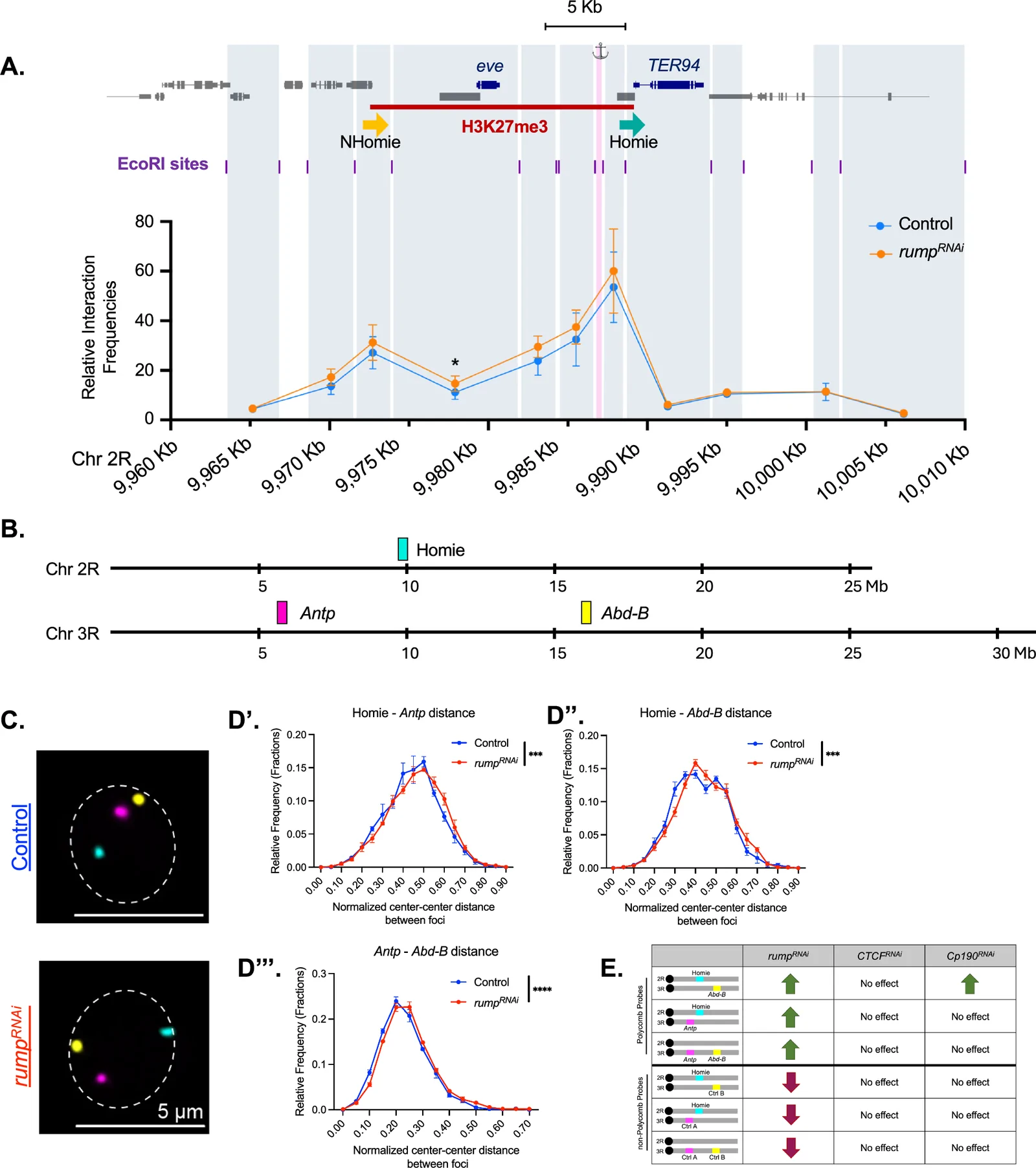
Rump alters both *cis* and *trans* Polycomb interactions. **A** 3C analysis of the *eve-TER94* locus after Rump depletion in Kc167 cells, using a region ~ 1.6 Kb upstream of Homie within the H3K27me3 as the anchor (pink). EcoRI restriction sites are shown in purple. The NHomie and Homie regions are marked by yellow and teal arrows, respectively. Interaction frequencies are shown on the y-axis, with the error bars indicating the standard deviation of the mean, and genomic coordinates are shown on the x-axis with a scale bar representing 5 Kb (n = 3 biological replicates; *, *p* < 0.05, Student’s t-test). **B** Schematic of the locations of Polycomb domain probes used in the Oligopaint DNA FISH analysis. The Homie probe is on Chr2R (teal), and the *Antp* (magenta) and *Abd-B* (yellow) probes are on Chr3R. **C** Representative images of Oligopaint DNA FISH probes in a control (top) or *rump*^*RNAi*^ Kc167 cell. Scale bar represents 5 µm. **D** Histograms showing relative frequency and center-center distance between foci normalized to cell size. Pairwise distances between Homie and *Antp* (top left), Homie and *Abd-B* (top right), and *Antp* to *Abd-B* (bottom) are shown for both control (blue) and *rump*^*RNAi*^ (red) nuclei (n = 3 biological replicates of > 1000 cells each; ****, *p* < 0.0001, ***, *p* < 0.001, Kolmogorov–Smirnov test). **E** Table summarizing all Oligopaint DNA FISH results for Polycomb and non-Polycomb domain distances after Rump, CTCF, or CP190 depletion.

Having observed changes in chromatin association of the PcG proteins as well as slight spreading of H3K27me3 onto *TER94* after Rump depletion, we wanted to determine whether 3D compaction of the *eve* Polycomb domain is also affected. To investigate this possibility, we performed circular chromatin conformation capture (3C) at the *eve* locus upon *rump* knockdown in Kc167 cells and assayed interaction frequencies between an anchor region 1.6 Kb upstream of Homie with surrounding upstream and downstream regions up to ~ 40 Kb. After verifying successful knockdown of Rump by Western blotting (Additional Fig. 11A), we found a slight increase in chromatin interaction frequencies compared to control cells (Fig. 5A and Additional Fig. 11B). Increased interactions were observed specifically within the *eve* Polycomb domain and at the Homie sequence, with no change in interaction occurring downstream of Homie and the *TER94* promoter. We confirmed these results by performing Hi-C, where uniformly increased interactions within the *eve* Polycomb domain are observed in Rump depleted compared to control cells (Additional Fig. 11C). As expected, higher intradomain interactions were observed for Polycomb versus similarly sized random domains genome-wide (Additional File 7 and Additional Fig. 11D). Importantly, a statistically significant increase is observed for all Polycomb intra-domain interactions compared to that of random domains in Rump depleted compared to control cells (Additional Fig. 11E). To test if increased compaction of the *eve* Polycomb domain was unique to Rump, we additionally performed 3C after depletion of either CTCF or CP190; however, we observed no change in interaction frequencies with any sites (Additional Fig. 12). These results suggest that depletion of Rump results in increased *cis-*compaction of Polycomb domains genome-wide including *eve* and that these effects occur independently of CTCF and CP190.

### Rump, but not insulator proteins, promotes 3D interactions between Polycomb domains *in trans*

With increased genome-wide Polycomb domain compaction and changes in PcG protein chromatin association after Rump depletion, we hypothesized that Rump depletion may also impact the global 3D organization of Polycomb domains. To investigate this possibility, we examined interactions between the *eve* Polycomb domain and other Polycomb domains *in trans* utilizing Oligopaint DNA FISH. We designed a 116 Kb Oligopaint DNA FISH probe on chr2R covering the 16 Kb *eve* Polycomb domain (Homie), which is the only Polycomb domain in this region. We also utilized two additional 174 and 195 Kb probes on chr3R covering 72% and 59% of the *Antp* and *Abd-B* Polycomb domains, respectively (Fig. 5B). These three probes were visualized simultaneously within control and Rump-depleted nuclei processed in parallel (Fig. 5C), allowing us to perform pairwise distance analyses in individual cells (n > 1000). Here, we observed across all comparisons that the distance between probes is slightly increased after Rump depletion (Figs. 5D’-D’’’, Additional Fig. 13 and Additional File 8), suggesting that 3D interactions between Polycomb domains are weakened. We also observed that this phenotype is Polycomb domain-specific by examining similarly sized probes covering non-Polycomb chromatin on chr3R (Fig. 5E and Additional Figs. 13 and 14). Interestingly, we observed that distances between the *eve* Polycomb domain and non-Polycomb regions are slightly decreased after Rump depletion (Additional Figs. 13–14). Importantly, we were able to confirm that interchromosomal Polycomb domain interactions were reduced after Rump depletion by analyzing Hi-C data generated from a control-*rump*^*RNAi*^ matrix. As expected, Polycomb domains interact more frequently with one another than Polycomb domains interact with a randomized set of domains (Additional Fig. 13A). Moreover, these Polycomb to Polycomb interactions were less frequent compared to Polycomb to random interactions in Rump depleted conditions compared to control cells (Additional Fig. 13B). Finally, we confirmed that the changes in Polycomb and non-Polycomb domain distances are unique to Rump depletion by performing Oligopaint DNA FISH after either CP190 or CTCF depletion. In these cases, we found that there is no change in any normalized probe distances with the exception of Homie to *Abd-B* distance being increased after CP190 depletion (Fig. 5E and Additional Fig. 15). Taken together, these results indicate that Rump but not insulator proteins regulate *trans* Polycomb domain organization, including interactions with the *eve* Polycomb domain.

## Discussion

**Fig. 6:**
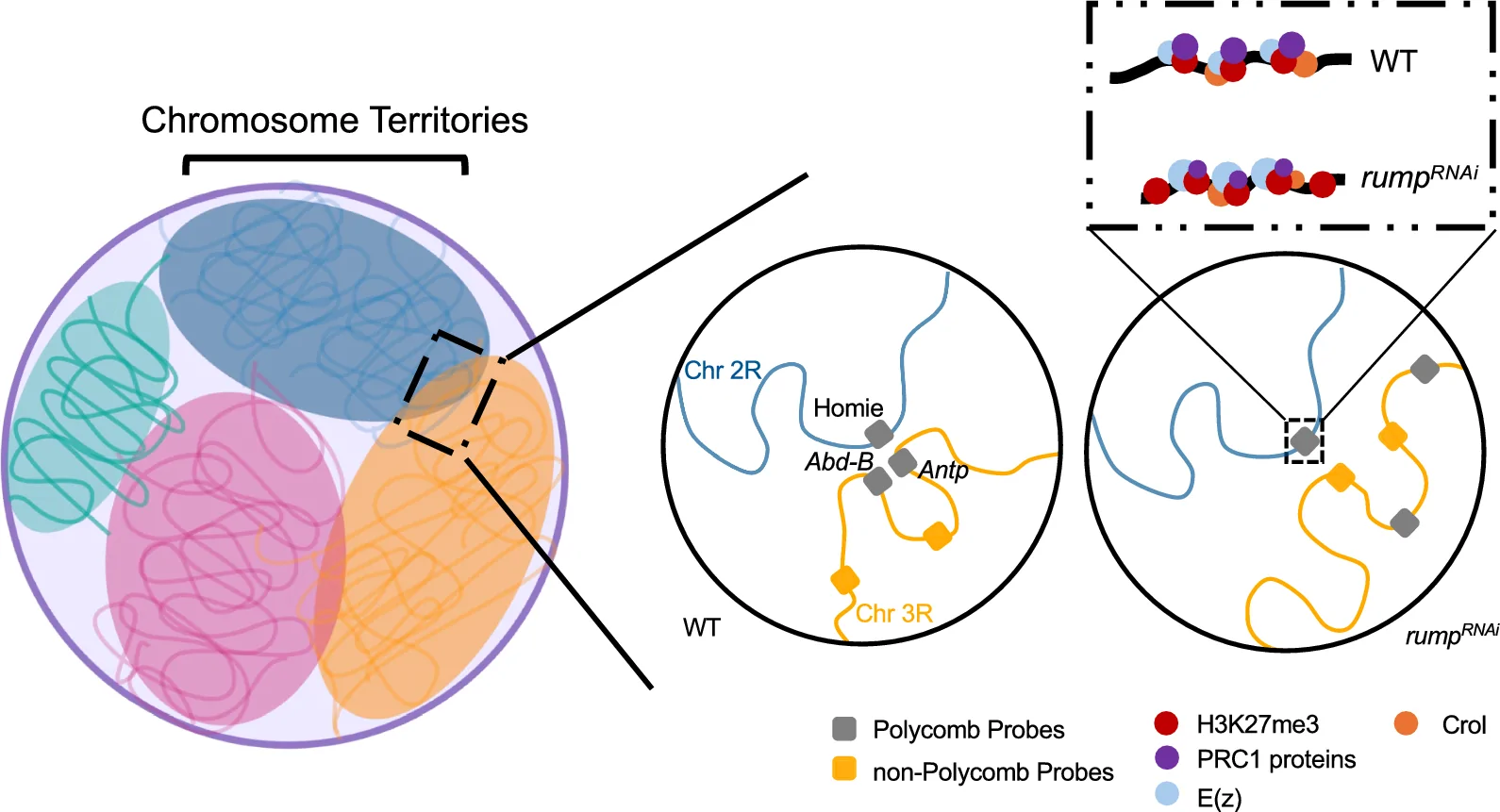
Summary of phenotypes observed after Rump depletion Schematic representing the primary phenotypes observed in Kc167 cells upon Rump depletion both *in cis* (at the *eve* Polycomb domain) and *in trans.*

Here we have demonstrated a novel role for Rump in the maintenance of 3D genome organization, specifically regulating both *cis* and *trans eve* Polycomb domain interactions. Using in vivo assays, we identified Rump as a *trans*-acting factor responsible for promoting Homie barrier activity. As Rump depletion results in *cis*-compaction of the *eve* domain and alters chromatin association of multiple PcG proteins, it is possible that promotion of Homie barrier activity by Rump occurs through effects on Polycomb domain formation *in cis* (Fig. 6). Specifically, binding of Crol was altered at 56% of Crol-bound PREs, including the PRE upstream of Homie. Chromatin association of E(z) was globally increased, coupled with spreading of H3K27me3 over Homie onto the *TER94* promoter, consistent with reduced *TER94* expression*.* Additionally, a global decrease in binding of all PRC1 proteins analyzed was observed, which might explain the reduction of 3D Polycomb domain interactions observed after Rump depletion (Fig. 6). Finally, we showed that Rump prevents *cis*-compaction of the *eve* domain. Interestingly, using our Homie barrier assay, we also identified CTCF and CP190 as *trans*-acting factors of Homie barrier activity; however, depletion of either CTCF or CP190 resulted in no change to *cis*-compaction or 3D Polycomb domain interactions. Together, these data highlight the importance of Rump in fine-tuning both *cis* and *trans* PcG interactions distinct from the activity of chromatin insulators.

### Role of Rump in maintaining the Homie barrier independently of chromatin insulator proteins

Using an overall protein depletion strategy, we showed that Homie barrier activity depends not only on Rump but also CTCF and CP190 but not Su(Hw). Interestingly, an alternative strategy of studying minimal sub-elements of the Homie insulator showed that a region containing a Su(Hw)-binding site is required and sufficient for Homie barrier activity [26]. Importantly, two additional non-overlapping sub-elements of Homie exhibited strong barrier activity in their assay. As Su(Hw), CTCF, and additional direct DNA-binding proteins are known to recruit CP190 to insulator sequences [13, 21–23, 55], it will be informative to explore how individual insulator protein recruitment to Homie sub-elements is achieved and how potential functional redundancy is built into this complex insulator sequence.

We observed that Rump depletion affects the *eve* Polycomb domain in a manner distinct from CTCF or CP190 depletion. Using 3C, we observed increased *cis*-compaction within the *eve* Polycomb domain and spreading of H3K27me3 past Homie onto the *TER94* promoter after Rump depletion. Consistent with the increase of H3K27me3, we observed a subtle increase of the PRC2 primary methyltransferase, E(z), near the Homie sequence. Perhaps the increase of E(z) and H3K27me3 upon Rump depletion creates stiffer or more compact chromatin at the *eve* locus, possibly limiting the ability of Homie to loop with NHomie, thus creating a weaker barrier. The ability of Homie and NHomie to loop are an important function of the Homie insulator as looping reinforces the *eve* TAD and isolates the *cis*-regulatory elements of *eve* [17–19]. Interestingly, we found that depletion of CTCF or CP190 does not affect *cis*-compaction of the *eve* Polycomb domain, and chromatin association of insulator proteins is unchanged at Homie after Rump depletion. These results are consistent with the possibility that Rump controls *eve* Polycomb domain compaction independently of chromatin insulators.

### The role of Rump in Polycomb formation

Our work suggests that Rump regulates chromatin association of PcG proteins during each step of Polycomb domain formation. The initial stage of Polycomb domain formation involves the recruitment of PcG proteins by PREs. We observe that Crol binding increases at 7% and decreases at 49% of Crol-bound PREs after Rump depletion. While Crol binding is not altered at all Crol-bound PREs, the reduction in Crol binding at the PRE upstream of Homie is suggestive of a mechanistic link between Crol loss and compromised Homie barrier activity. This effect of Rump depletion is unique to Crol binding; we observed that two additional PRE-associated proteins, Pho and Cg, show little to no altered chromatin association at PRE sites. However, we noted at nonPRE sites, Pho and Cg signal mimics that of the differential Crol binding sites with which they overlap. Our results are consistent with a previous study that implicated Crol in recruitment of Cg to nonPRE chromatin regions [52], and suggest that Crol may help to recruit other PRE associated proteins, such as Pho, to nonPRE chromatin.

In addition to changes in Crol chromatin binding, we also observe global chromatin association changes of both PRC1 and the primary PRC2 methyltransferase, E(z). In both contexts the changes are subtle as we observe no significant changes by DiffBind analysis. However, metaplots of the signal especially at PREs show a moderate decrease or increase in chromatin association upon Rump depletion for PRC1 and E(z), respectively. Importantly, both PRC1 and PRC2 are recruited to chromatin in part by PRE-associated proteins, of which Crol is only one of many. Given the redundancy in PRE-associated proteins, it is likely that reduction of one PRE-associated protein at only a subset of PRE sites is not enough to substantially alter chromatin association of PRC1 or PRC2. Therefore, we observe only subtle decreases in chromatin association upon Rump depletion. Finally, as there is less PRC1 bound to these sites, this effect might stochastically allow for more binding of E(z) as detected in our experiments. Together, these data suggest a role of Rump in fine-tuning the homeostatic conditions of PcG regulation, potentially through proper regulation and recruitment of Crol.

### Role of Rump in Polycomb-dependent 3D interactions

Our work highlights that Rump depletion not only alters Polycomb domain formation and maintenance *in cis* but also impacts 3D PcG interactions *in trans.* Rump depletion resulted in increased distances between all three Polycomb domains analyzed and a decrease in Hi-C interaction frequency between Polycomb domains genome-wide. These data implicate Rump as a novel regulator of 3D Polycomb domain interactions. Interestingly, an RNAi screen determined that Crol knockdown led to weaker and more diffuse Pc foci [56]. Moreover, reduced chromatin association of PRC1, of which the SAM domain in Ph was specifically shown to mediate PcG clustering [9], could further explain reduced 3D Polycomb domain interactions after Rump depletion. Importantly, these effects on 3D genome organization after Rump depletion appear Polycomb domain-specific since distances between two non-Polycomb domains instead decrease. Finally, we again observed that depletion of insulator proteins did not systematically affect Polycomb domain *trans* interactions, emphasizing a specific role for Rump in maintaining 3D Polycomb domain interactions.

## Conclusions

In summary, we identified a role for Rump in promoting Homie barrier activity, and its function in this capacity is likely independent of chromatin insulator proteins. Rump affects the *cis*-recruitment of certain PcG proteins to chromatin and also regulates Polycomb domain 3D interactions. Further studies will be required to address the molecular details by which Rump regulates 3D genome organization.

## Supplementary Information

Supplementary Material 1. **Additional Figure 1**: *eve-TER94 *locus in Kc167 cells. Screenshot showing the wild-type chromatin association of H3K27me3, the *gypsy *core complex (Su(Hw), Mod(mdg4)67.2, and CP190), CTCF, Rump, PRC1 components (Pc, Psc, and Ph), PRC2 components (E(z) and Su(z)12), and PRE-associated proteins (Crol, Pho, and Cg) at the *eve-TER94* locus. Additionally, RNA-seq from wild-type Kc167 cells is shown. **Additional Figure 2**: *TER94 *expression is downregulated in *rump *mutant embryos and *rump*^*RNAi*^ Kc167 cells. A. RT-qPCR for *GFP *mRNA expression in embryos expressing a *TER94-GFP *transgene normalized to housekeeping gene *RpL35*. Bar graph represents the mean of *GFP *expression with standard deviation represented by error bars (n= 3 biological replicates; **** p <0.0001, Student’s t-test). B. RT-qPCR for *rump *mRNA expression in embryos expressing a *TER94-GFP* transgene normalized to housekeeping gene *RpL35*. Bar graph represents the mean of rump expression with standard deviation represented by error bars (n= 3 biological replicates; **** p <0.0001, Student’s t-test). C. Volcano plot of differentially expressed genes identified in RNA-seq after Rump depletion. The -log(FDR) is shown on the y-axis and a gray dotted line indicates a -log(FDR) cutoff of 1.3 (p-adjusted value of 0.05). In light orange are genes with a -log(FDR) above the cutoff. Note that rump expression (labeled red dot) shows a large increase due to the presence of dsRNA used for knockdown. **Additional Figure 3**: Homie-Luciferase plasmid map and insertion location at *attp3 *locus on Chromosome X. Map of the Homie-Luciferase plasmid inserted into the *attp3 *locus on chromosome X (top). The luciferase gene (yellow) is driven by the hsp70 promoter (green arrow) with 5x UAS sequence (turquoise square) followed by the Sv40 polyA sequence (blue arrow). The entire sequence is flanked upstream and downstream with the Homie sequence (teal box). Additionally, a screenshot of the *attp3 *locus in chromosome X is shown (bottom). The screenshot contains TAD domain calls from embryo and Kc167 data showing that the region is repressed. Finally, ChIP-seq tracks of CP190 and CTCF are shown in Kc167 cells, embryo, and larval CNS tissue. **Additional Figure 4**: Confirmation of Barrier Assay. A. Schematic of all luciferase barrier assay constructs. UAS-Luciferase transgenes are uninsulated (top left), flanked by Homie sequences in its forward orientation (Homie-insulated, top right), flanked by the *gypsy *insulator sequence (gypsy-insulated, bottom left), or flanked by the Homie sequence in its endogenous (teal arrow) and reverse (blue arrow) orientations (Homie-rev-insulated, bottom right). B. Box-and-whisker plots of luciferase assay using uninsulated, *gypsy*-insulated, Homie-insulated, or Homie-rev-insulated lines. A ubiquitous (Act5C, left), muscle-specific (Mef2, center) or central nervous system-enriched (l(3)31-1, right) driver was used. Relative luciferase is normalized to total protein (n = 12 larvae per genotype for Act5C and Mef2, n = 12 sets of 3 male larval brains per genotype for l(3)31-1; ****, p < 0.0001, **, p < 0.005, one-way ANOVA followed by Tukey post hoc test). The median is indicated by a line between the lower and upper quartiles, while the whiskers represent the min and max of each data set. C. Western blots showing knockdown efficiency of CP190 (top left), CTCF (top right), Su(Hw) (bottom left), or Rump (bottom right) depletions in Uninsulated, *gypsy*-insulated, Homie-insulated, or Homie-rev-insulated third-instar larvae after RNAi using da-GAL4 driver for CTCF, Su(Hw), or Rump. Mef2-GAL4 driver was used for CP190 depletion. For CP190 and CTCF, α-Fibrillarin is used as a loading control, and for Su(Hw) and Rump, α-Tubulin is used as loading control. **Additional Figure 5**: Depletion of Rump leads to mild changes in *crol *mRNA levels while steady state protein levels appear unaffected. A. RNA-seq track showing the crol locus in Kc167 cells in control (top) and *rumpRNAi *(bottom) conditions. *crol *is downregulated after Rump depletion (log2FC: -0.32, padj: 0.0031). Red bar indicates a zoomed in region (in B) of the 5’ UTR that is alternatively spliced. B. Sashimi plots (orientation flipped relative to (A)) showing an aberrant skipped-exon occurring in the 5’ UTR of the *crol *transcript after Rump depletion. Splicing events are shown both in control (top) and *rump*^*RNAi*^ (bottom) in Kc167 cells with RPKM and genomic location shown. The inclusion level (IncLevel) for each biological replicate is shown on the right side of the signal track, and a schematic of the 5’ exons including an alternatively skipped exon is shown beneath the tracks (in black) (padj: 1.2e-05). C. Western blot showing steady-state Crol protein levels in both control and *rump*^*RNAi*^ conditions in Kc167 cells. CP190 protein is used as a loading control (n=6 biological replicates). **Additional Figure 6**: Additional analysis of Crol binding sites. A. Screenshot of Crol ChIP-seq in control (gray tracks) and *rump*^*RNAi*^ (orange tracks) conditions. Merged ChIP-seq signal as well as the three biological replicates are shown. Crol peaks in control conditions are shown in gray while downregulated and upregulated binding sites, identified by DiffBind analysis, are shown in red and blue, respectively. B. Heatmap showing average Crol ChIP-seq signal plotted +/- 1.5 Kb of peak center at PRE sites and nonPRE Crol binding sites. C. Heatmap showing average Crol ChIP-seq signal plotted +/- 1.5 Kb of peak center at nonPRE Crol sites that are unchanged, upregulated, or downregulated after Rump depletion. D. Heatmap showing average Crol ChIP-seq signal plotted +/- 1.5 Kb of peak center at Crol-bound PRE sites that are unchanged, upregulated, or downregulated after Rump depletion. **Additional Figure 7**: Depletion of Rump leads to changes in *cg *RNA levels and *pho *alternative splicing. A. RNA-seq track showing the *cg *locus in Kc167 cells in control (top) and *rump*^*RNAi*^ (bottom) conditions. *cg *is mildly downregulated after Rump depletion (log2FC: -0.20, padj:0.044). B. RNA-seq track showing the *pho *locus in Kc167 cells in control (top) and *rumpRNAi *(bottom) conditions. *pho *levels are not significantly altered after Rump depletion (log2FC: -0.033, padj:0.87). Red bar indicates a zoomed in region (in C) of the 5’ UTR that is alternatively spliced. C. Sashimi plots (orientation flipped relative to (B)) showing 2 splicing events in the 5’ UTR of the *pho *transcript that are more frequent after Rump depletion. Splicing events are shown both in control (top) and *rump*^*RNAi*^ (bottom) in Kc167 cells with RPKM and genomic location shown. The inclusion level (IncLevel) for each biological replicate is shown on the right side of the signal track, and a schematic of the splicing events is shown beneath the tracks (in black) (padj: 0.033 (left) and padj: 0.002 (right)). **Additional Figure 8**: Chromatin association of certain PRE-binding, PRC1, and PRC2 proteins show few changes after Rump depletion. MA-plots showing differential H3K27me3, PRC1 (Pc, Psc, or Ph), PRC2 (E(z) or Su(z)12), or PRE-binding proteins (Pho or Cg) peaks after Rump depletion. Plotted on the y-axis is log2FC (*rump*^*RNAi*^-Control) and plotted on the x-axis is log2Concentration using DESeq2:RLE normalization. Differential peaks (represented by pink dots) are identified using a padj cutoff of 0.05. **Additional Figure 9**: Heatmaps of PRC1, PRC2, Pho, and Cg signal at Crol differential and non-differential sites after Rump depletion. A. Heatmaps showing average PRC1, PRC2, Pho, and Cg ChIP-seq signal plotted +/- 1.5 Kb of peak center at nonPRE Crol sites that are unchanged, upregulated, or downregulated after Rump depletion. B. Heatmaps showing average PRC1, PRC2, Pho, and Cg ChIP-seq signal plotted +/- 1.5 Kb of peak center at nonCrol PRE sites and Crol-bound PRE sites that are unchanged, upregulated, or downregulated after Rump depletion. **Additional Figure 10**: Metaplots of H3K27me3 signal at *eve*, ANTP, and BX-C loci by replicate. A-C. H3K27me3 (at the *eve *(A), *Antp *(B), or BX-C (C) domains) ChIP-seq signal plotted +/- 2 Kb of H3K27me3 domain peak boundaries in control conditions. Each biological replicate is shown as an individual plot comparing control to *rump*^*RNAi*^ conditions. **Additional Figure 11**: Rump depletion leads to significant increased *cis*-compaction at the *eve *Polycomb domain and increased compaction at all Polycomb domains globally. A. Western blot showing knockdown efficiency of Rump depletions in Kc167 cells with α-Tubulin used as loading control. B. The same 3C analysis of the *eve-TER94* locus in Kc167 cells after Rump depletion data as shown in Figure 5A but represented as individual replicates. Interaction frequencies are shown on the y-axis and genomic coordinates are shown on the x-axis (n= 3 biological replicates; *, p < 0.05, Student’s t-test). C. Hi-C matrix showing *rump*^*RNAi*^ contacts/Control contacts at the *eve-TER94* locus in Kc167 cells at 1 Kb resolution where red signifies increased contacts in *rump*^*RNAi*^ and blue signifies increased contacts in the control. The *eve *Polycomb domain is denoted with a red line, with the Homie and NHomie regions marked by teal and yellow arrows, respectively. D. Violin plots showing observed/expected (O/E) intra-domain interactions in Polycomb (pink) or similarly sized randomized regions of chromatin (gray) in both control and Rump depleted conditions calculated from a 5 Kb Hi-C matrix (n >233 interactions, ****, p <0.001, Student’s t-test). Values >1 indicate higher interaction than expected by chance while values <1 indicate lower interaction than expected by chance (y=1 is marked with a dotted gray line). The median is indicated by a dashed line between the lower and upper quartiles (dotted line). E. Histogram showing observed/expected (O/E) intra-domain interactions in Polycomb (pink) or similarly sized randomized regions of chromatin (gray) calculated from a 5 Kb Control-*rump*^*RNAi*^ Hi-C matrix (n >233 interactions, ****, p <0.001, Kolmogorov–Smirnov test). Negative values indicate higher interactions in Rump depleted conditions while positive values indicate higher interactions in control conditions. **Additional Figure 12**: Neither CTCF nor CP190 alter cis-compaction at the *eve-TER94* locus. A-B. Western blots showing knockdown efficiency of CTCF (A) or CP190 (B) depletions in Kc167 cells with α-Tubulin used as loading control. C-D. 3C analysis of the *eve-TER94 *locus in Kc167 cells after either CTCF (C) or CP190 (D) depletion, using a region ~1.6 Kb upstream of Homie within the H3K27me3 domain as the anchor (pink). EcoRI restriction sites are shown in purple. The Homie and NHomie regions are marked by a teal and yellow arrow, respectively. Interaction frequencies are shown with error bars indicating standard deviation of the mean, and genomic coordinates are shown with a scale bar representing 5 Kb (n= 3 biological replicates; *, p < 0.05, Student’s t-test). **Additional Figure 13**: Polycomb domain interactions are reduced, and Polycomb domain probe distances are increased after Rump depletion. A. Violin plots showing raw observed interchromosomal interactions between Polycomb domains (pink) or interactions between Polycomb domains with randomized regions of chromatin (gray) in both control and Rump depleted conditions calculated from a 25 Kb Hi-C matrix (n >233 interactions, ****, p <0.001, Student’s t-test). The median is indicated by a dashed line between the lower and upper quartiles (dotted line). B. Histogram showing raw observed interchromosomal interactions between Polycomb domains (pink) or interactions between Polycomb domains with randomized regions of chromatin (gray) calculated from a 25 Kb Control-*rump*^*RNAi*^ Hi-C matrix (n >233 interactions, ****, p <0.001, Kolmogorov–Smirnov test). Negative values indicate higher interactions in Rump depleted conditions while positive values indicate higher interactions in control conditions. C-D. Normalized center-center distances between Polycomb foci and non-Polycomb foci analyzed in Figure 5D and Additional Figure 14C, respectively, were additionally visualized using violin plots showing the median (dashed line) and upper and lower quartiles (dotted line) (B) or cumulative frequency distribution plots (C). **Additional Figure 14**: Non-Polycomb domain probes are closer together after Rump depletion. A. Schematic of locations of the non-Polycomb domain probes used in the Oligopaint DNA FISH analysis. The Homie probe is on Chr2R (teal) and the Control A (magenta) and Control B (yellow) probes are on Chr3R. The Polycomb probes covering *Antp *and *Abd-B *are represented in gray. B. Representative images of Oligopaint DNA FISH probes in a control (top) or *rump*^*RNAi*^ Kc167 cell. Scale bar represents 5 µm. C. Histograms showing relative frequency and center-center distance between foci normalized to cell size. Pairwise distances between Homie and Control A (top left), Homie and Control B (top right), and Control A and Control B (bottom) are shown for both control (blue) and *rump*^*RNAi*^ (red) nuclei (n= 3 biological replicates of >1000 cells each; ****, p <0.0001, ***, p<0.001, Kolmogorov–Smirnov test). **Additional Figure 15**: CTCF and CP190 largely have no effect on inter-Polycomb/non-Polycomb probe distances. A. Histograms plotting relative frequency and center-center distance between foci normalized to cell size. Pairwise distances between Polycomb domains (top) and non-Polycomb domains (bottom) are shown for both control (blue) and *CTCF*^*RNAi*^ (lavender) nuclei (n= 3 biological replicates of >1000 cells each; all not significant, p-value >0.05, Kolmogorov–Smirnov test). B. Histograms plotting relative frequency and center-center distance between foci normalized to cell size. Pairwise distances between Polycomb domains (top) and non-Polycomb domains (bottom) are shown for both control (blue) and *Cp190*^*RNAi*^ (pink) nuclei (n= 3 biological replicates of >1000 cells each; **, p<0.005, Kolmogorov–Smirnov test).

Supplementary Material 2. Primer sequences used for luciferase barrier assays, RT-qPCR, and 3C assays.

Supplementary Material 3. DESeq2 results from RNA-seq after Rump depletion.

Supplementary Material 4. Luciferase barrier assay p-values for data in Figure 1 and Additional Figure 4B.

Supplementary Material 5. Differential binding results from all 13 factors analyzed by ChIP-seq after Rump depletion, ordered by appearance in the text.

Supplementary Material 6. Differential splicing analysis of RNA-seq data after Rump depletion.

Supplementary Material 7. Hi-C interaction values and data used to plot the graphs in Additional Figures 11 and 13.

Supplementary Material 8. Oligopaint DNA FISH probe regions and raw data used to plot the normalized graphs in Fig. 5 and Additional Figures 13-15

Supplementary Material 9. List of Antibodies used for western blots and ChIP-seq.

Supplementary Material 10. List of ChIP-seq input files uploaded to GEO with their corresponding IPs.

## Data Availability

Datasets used to generate the results of this study can be found in the supplementary information files of this publication. Additionally, RNA-seq, ChIP-seq, and Hi-C data are available in GEO under GSE308570 , GSE308569 , and GSE325266, respectively.
