# Supplementary material for: Drosophila Rumpelstiltskin regulates Polycomb cis and trans interactions and Homie barrier activity
Source: Epigenetics Chromatin. 2026 Aug 6;19:36. doi: 10.1186/s13072-026-00694-x (PMC13504762; doi:10.1186/s13072-026-00694-x)

Additional Figure 1: *eve-TER94* locus in Kc167 cells

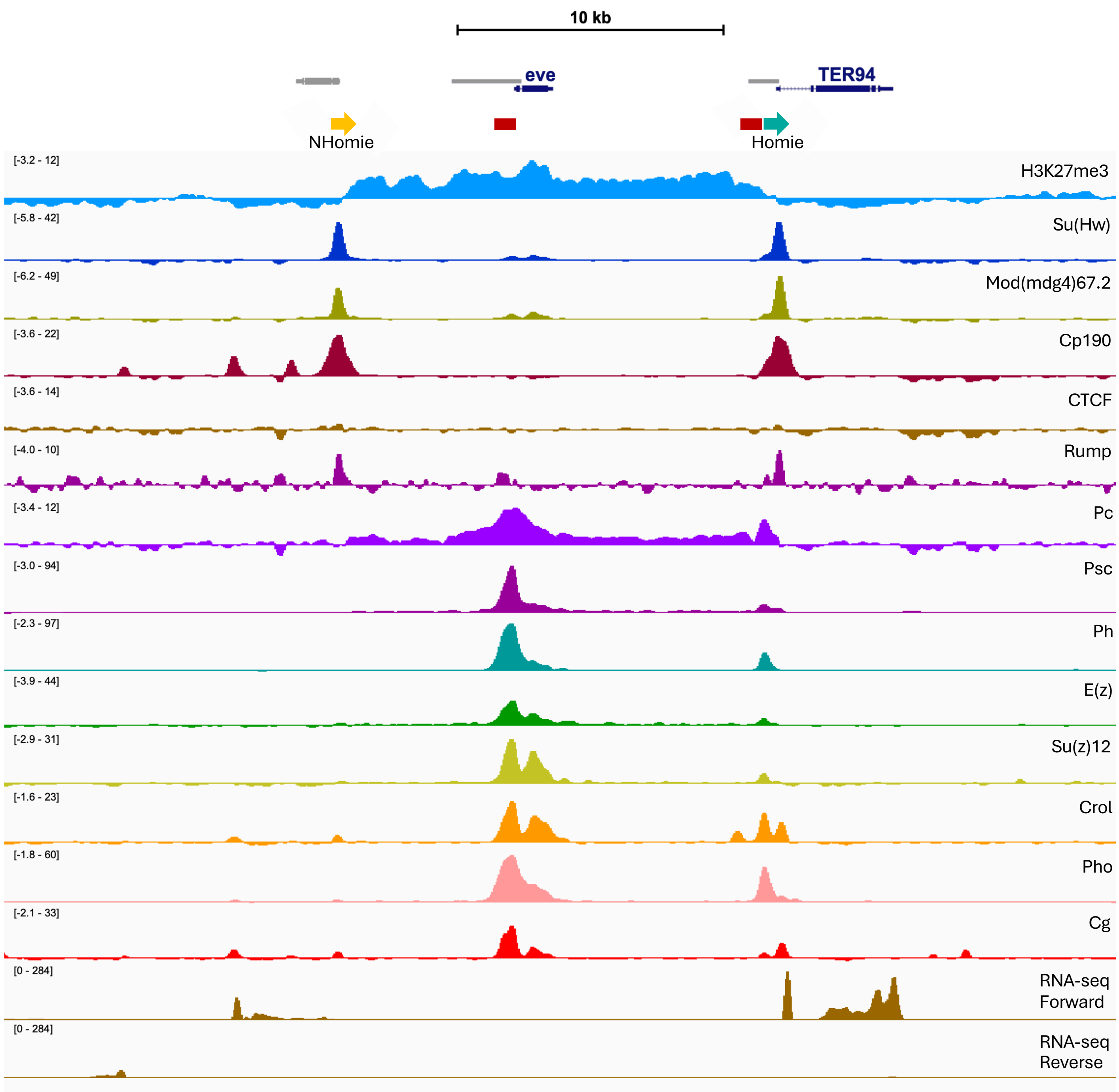

Additional Figure 2: *TER94* expression is downregulated in *rump* mutant embryos and *rump*^{RNAi} Kc167 cells.

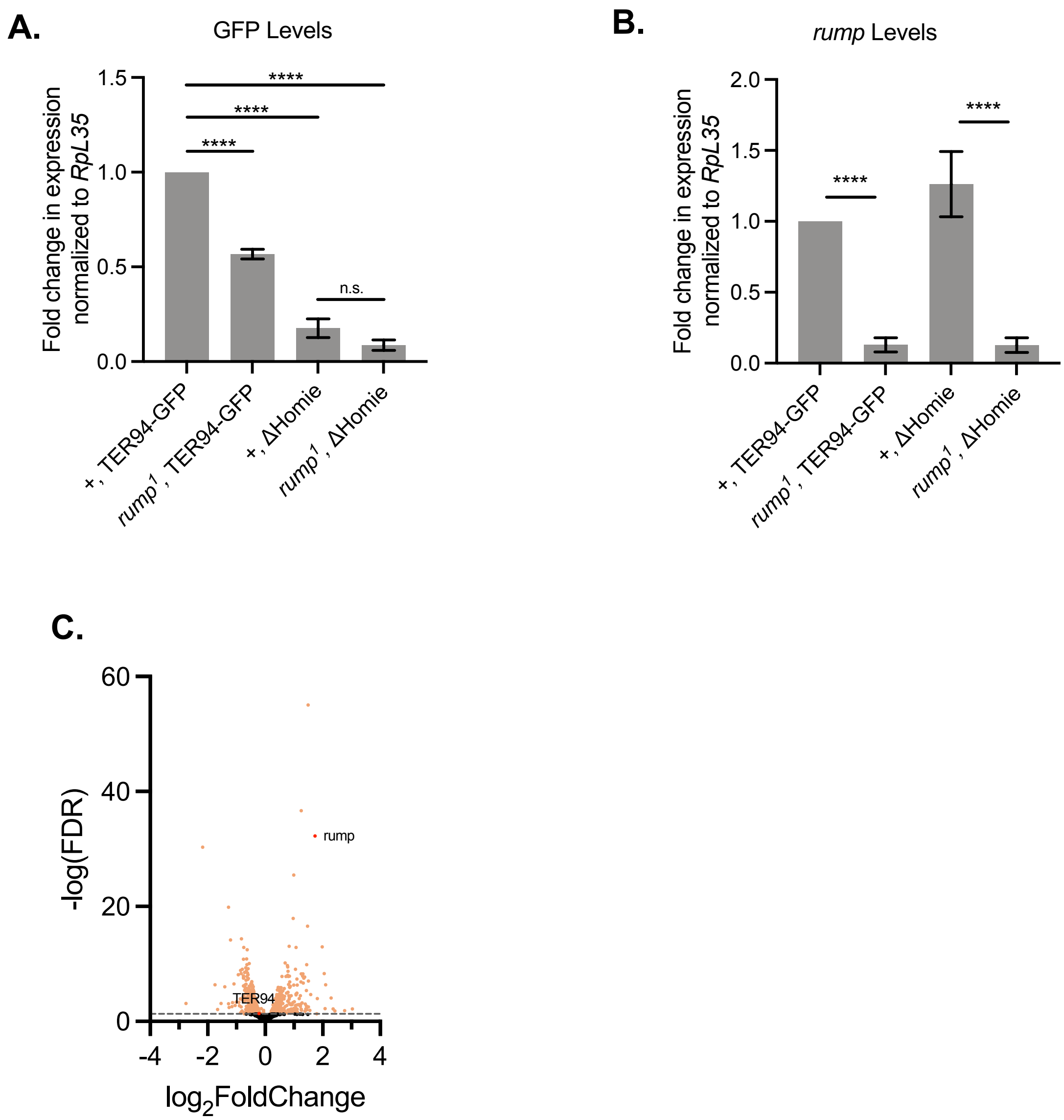

Additional Figure 3: Homie-Luciferase plasmid map and insertion location at *attp3* locus on Chromosome X.

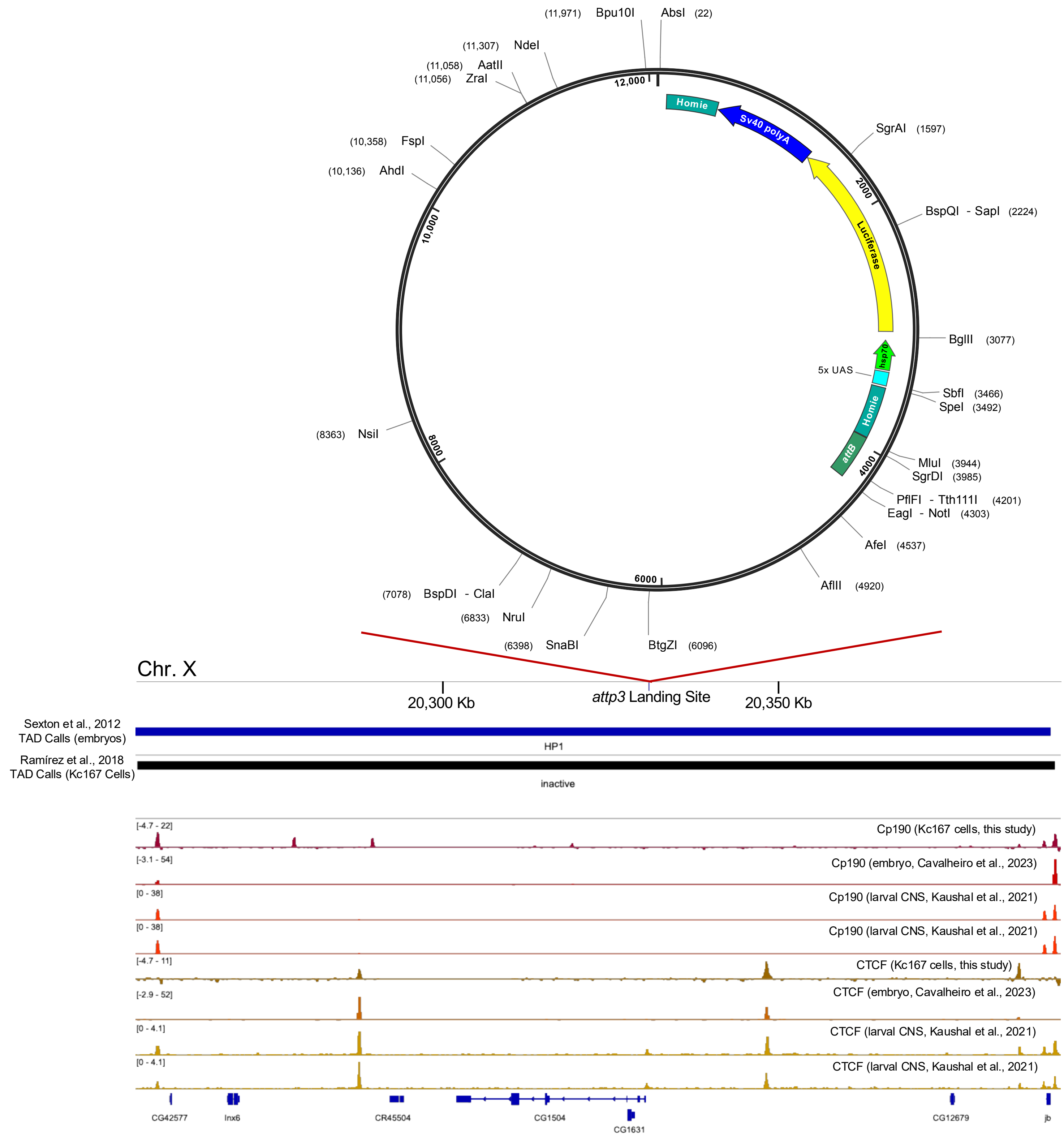

Additional Figure 4: Confirmation of Barrier Assay

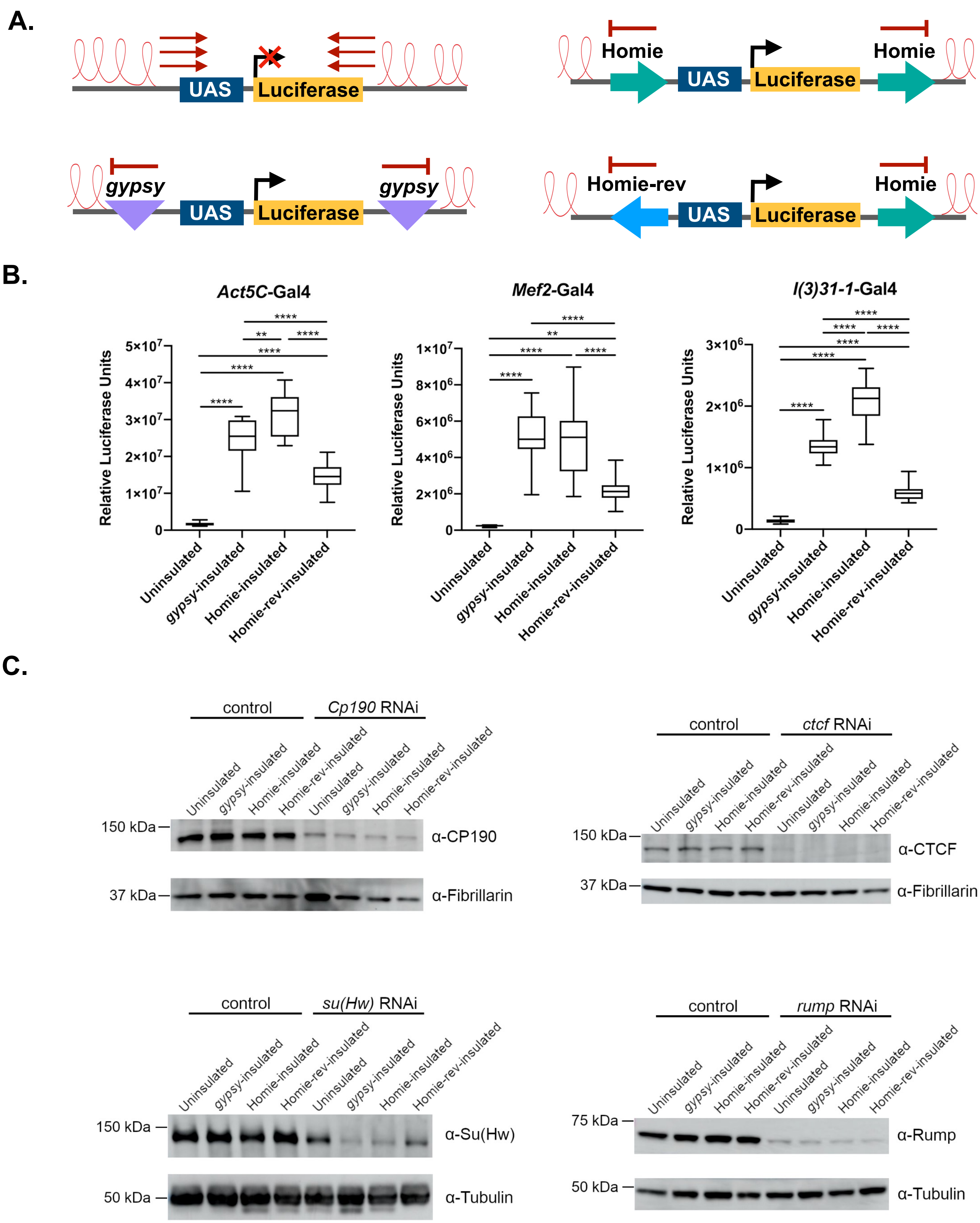

Additional Figure 5: Depletion of Rump leads to changes in *crol* mRNA levels while steady state protein levels appear unaffected.

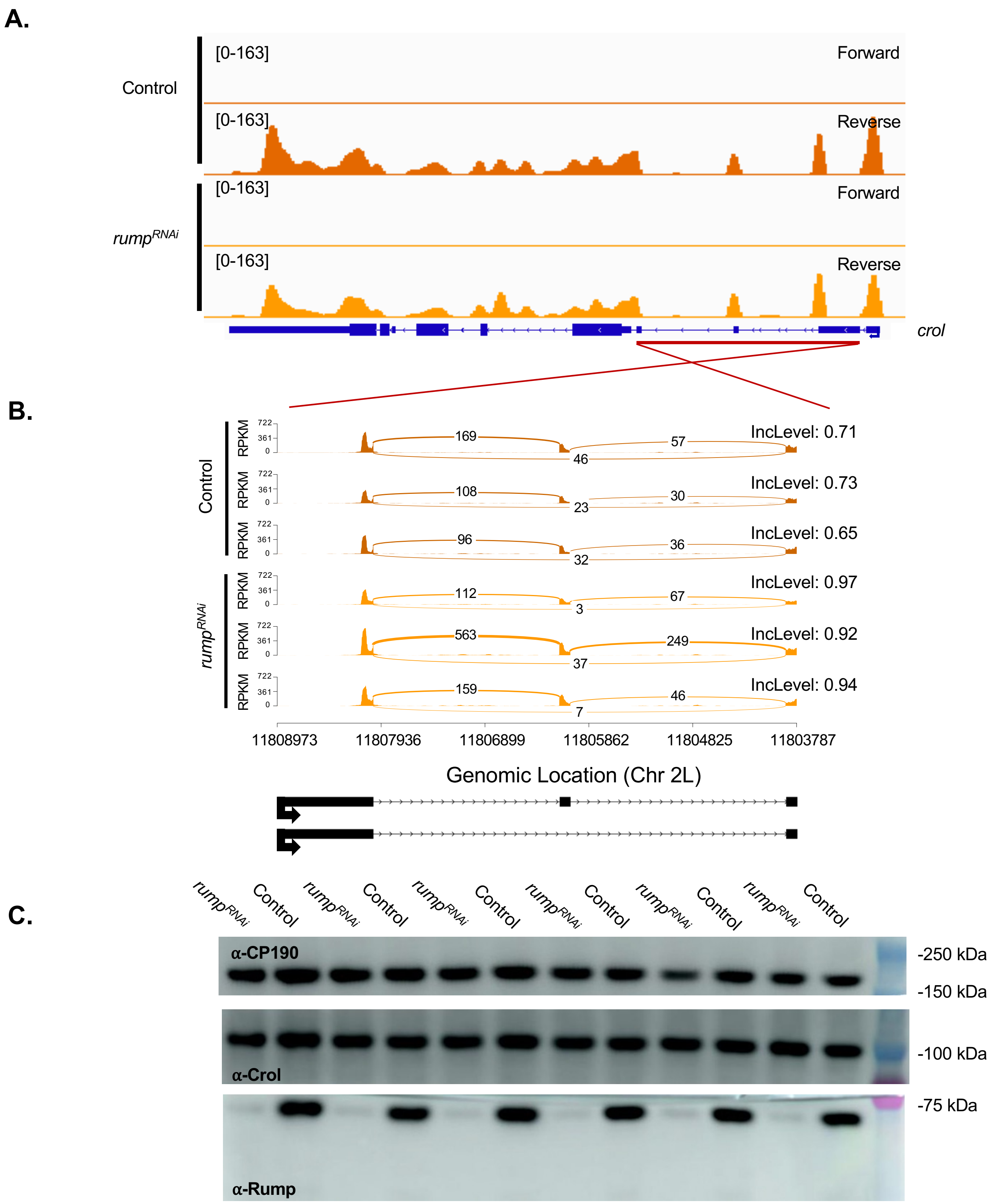

Additional Figure 6: Additional analysis of Crol binding sites

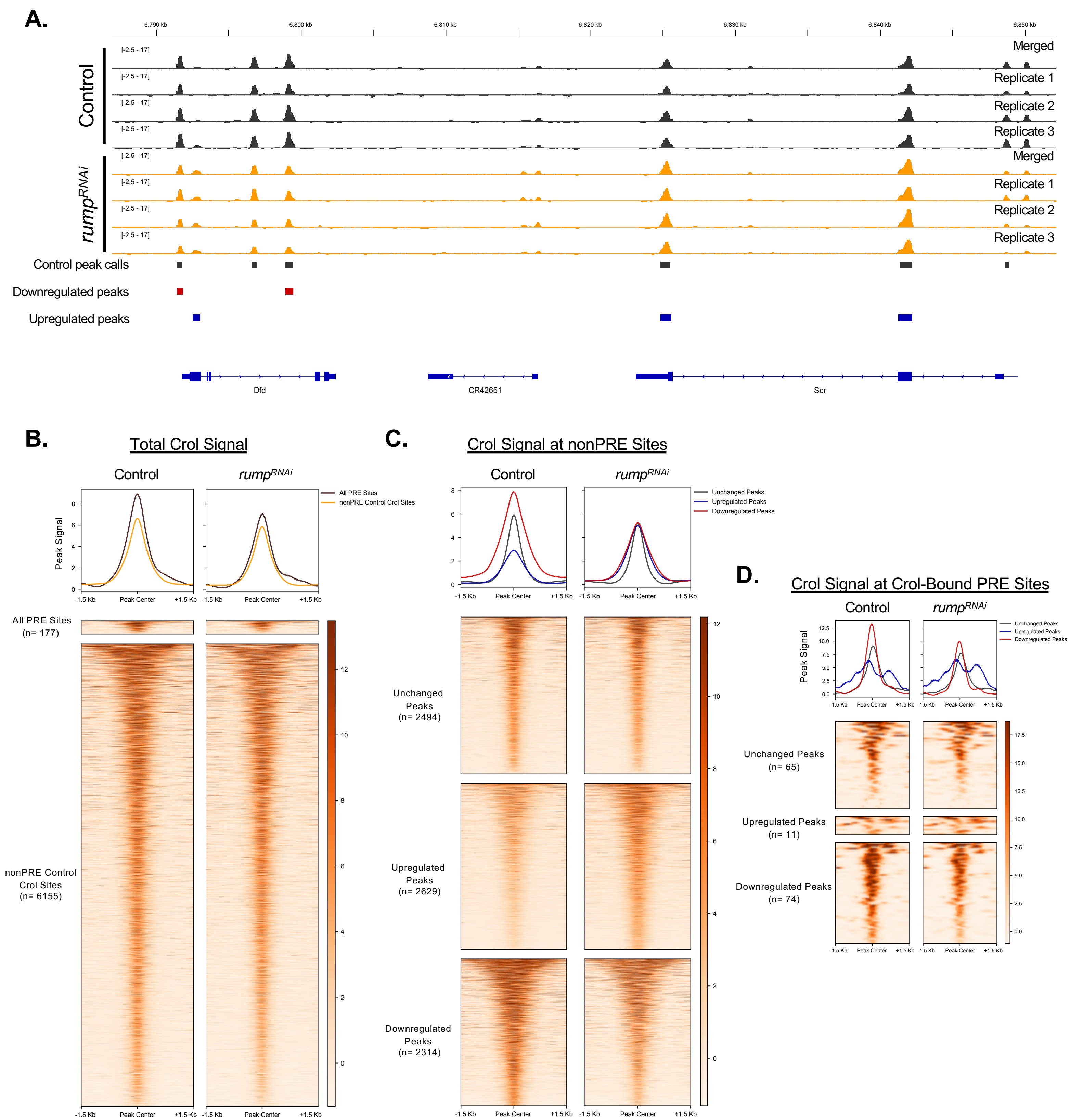

Additional Figure 7: Depletion of Rump leads to changes in *cg* RNA levels and *pho* alternative splicing.

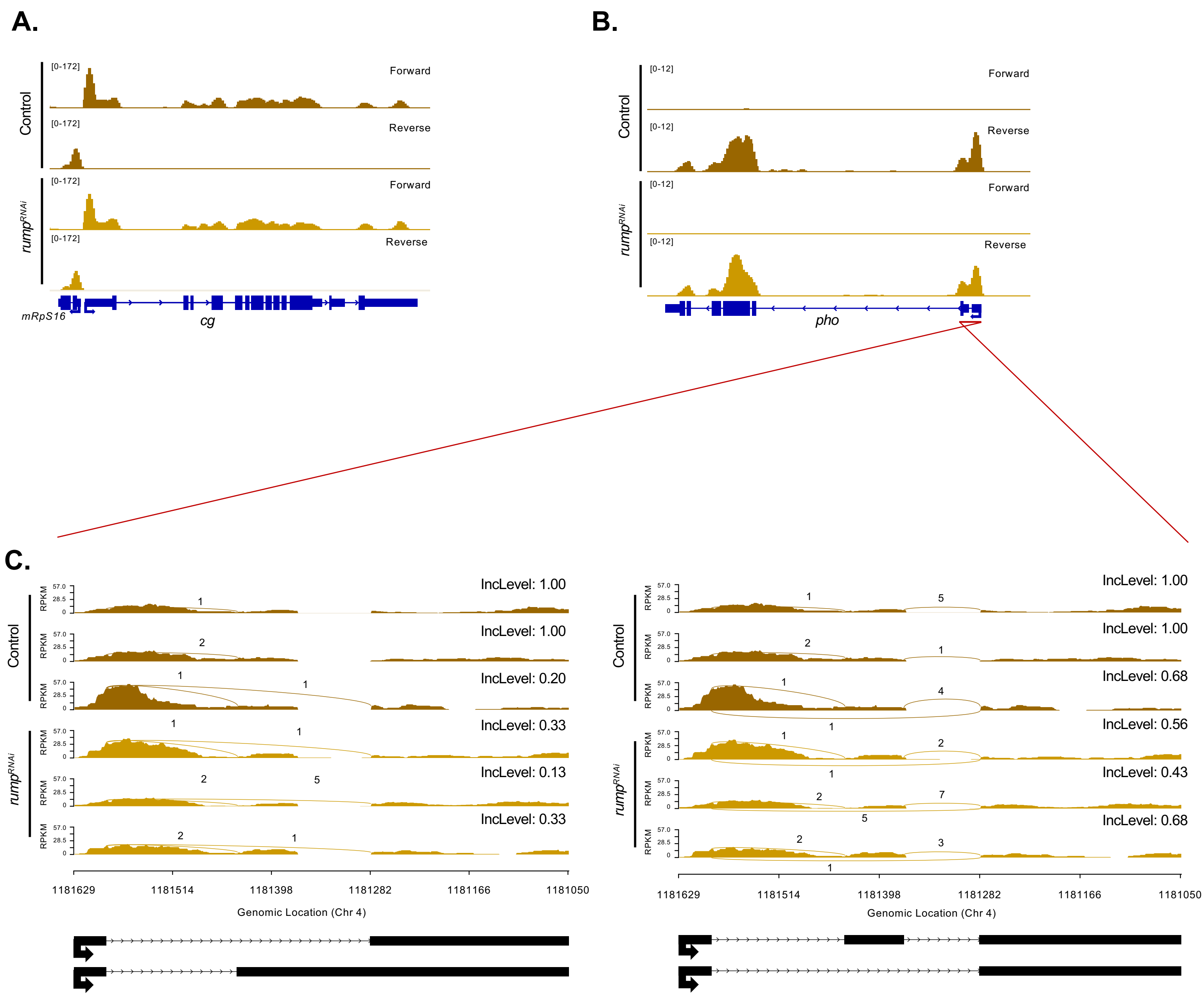

Additional Figure 8: Chromatin association of certain PRE-binding, PRC1, and PRC2 proteins show few changes after Rump depletion.

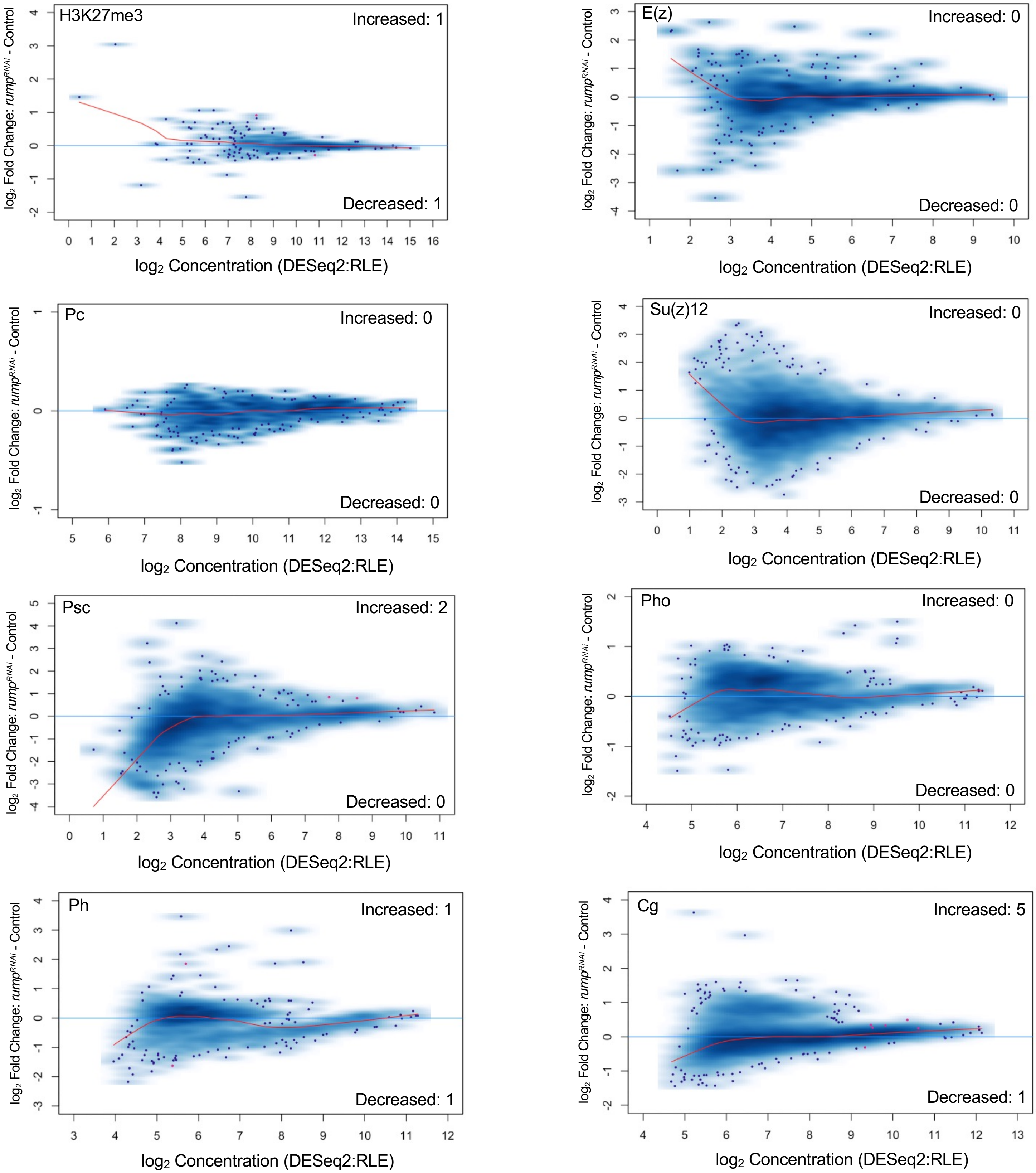

Additional Figure 9: Heatmaps of PRC1, PRC2, Pho, and Cg signal at CroI differential and non-differential sites after Rump depletion.

A.

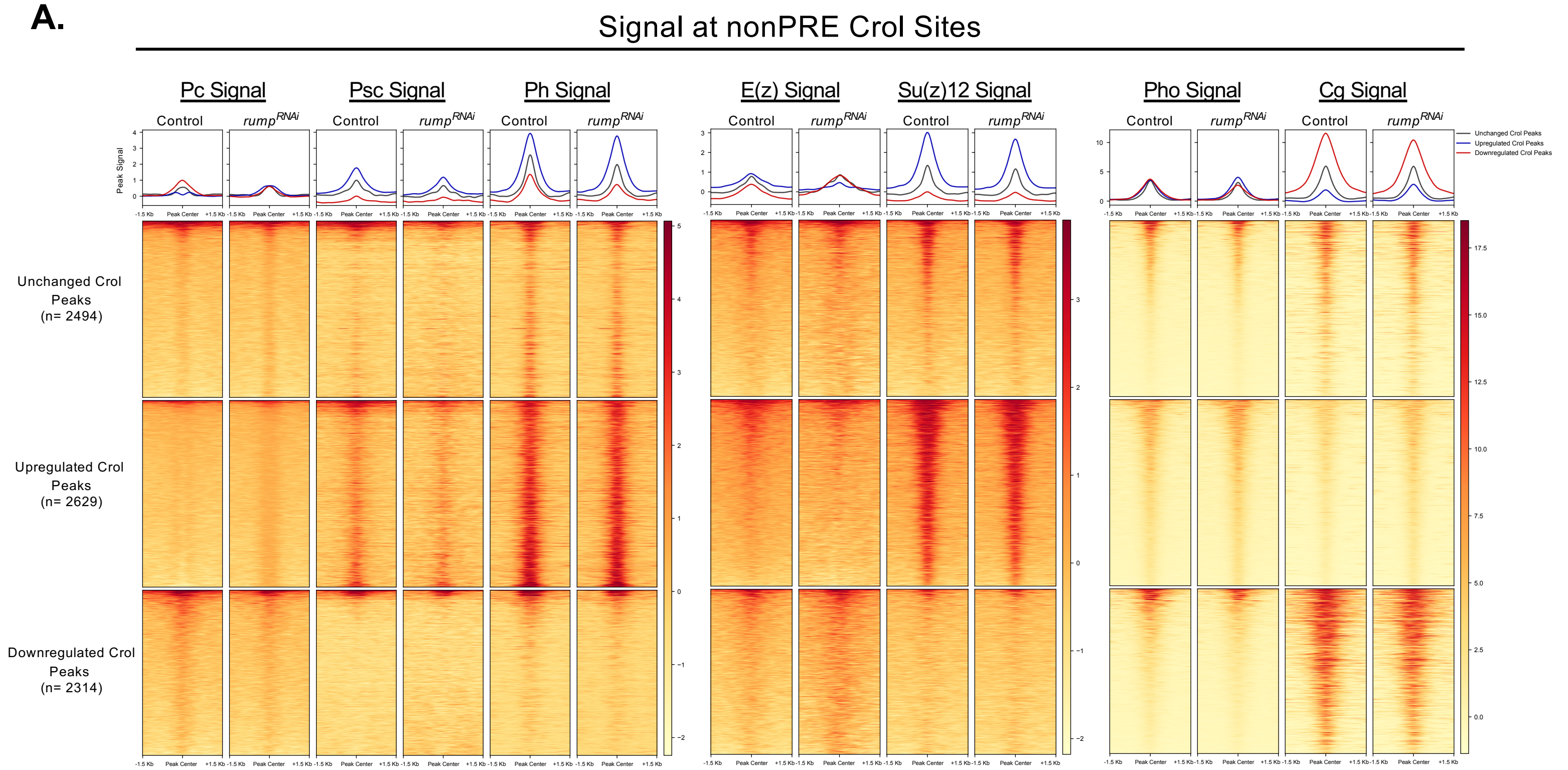

B.

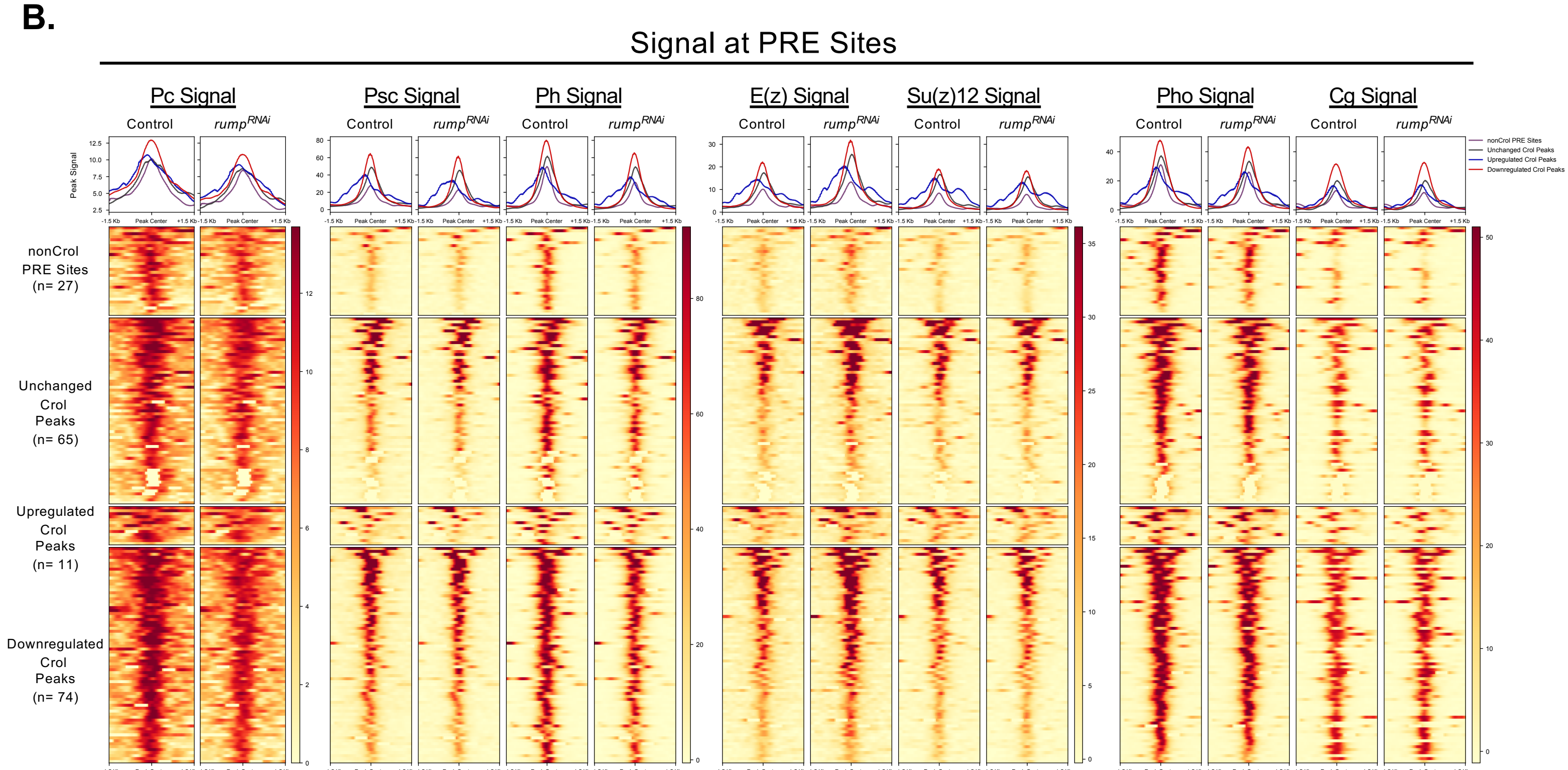

Additional Figure 10: Metaplots of H3K27me3 signal at *eve, ANTP,* and BX-C loci by replicate.

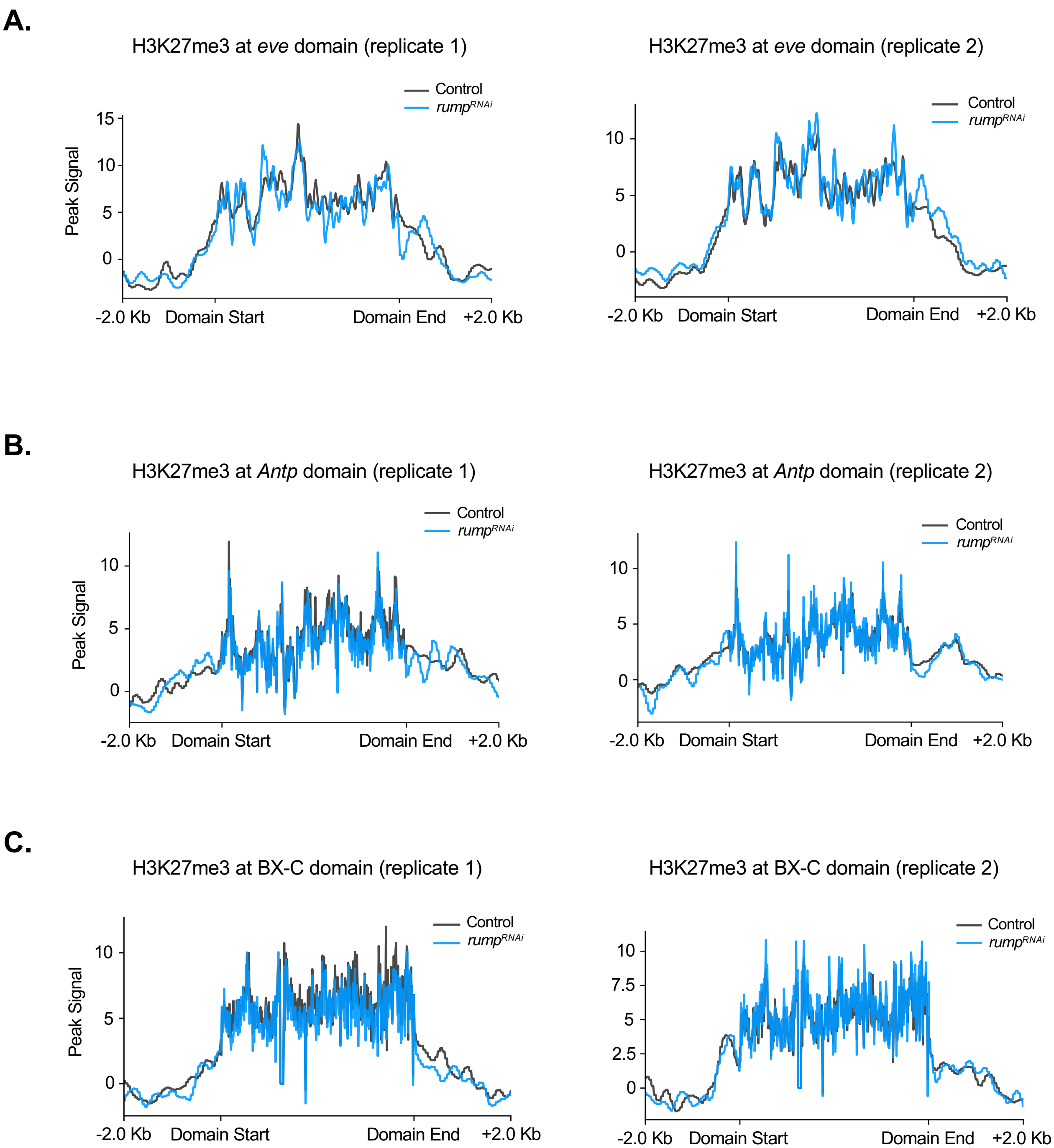

Additional Figure 11: Rump depletion leads to significant increased *cis*-compaction at the *eve* Polycomb domain and subtle increased compaction at all Polycomb domains globally.

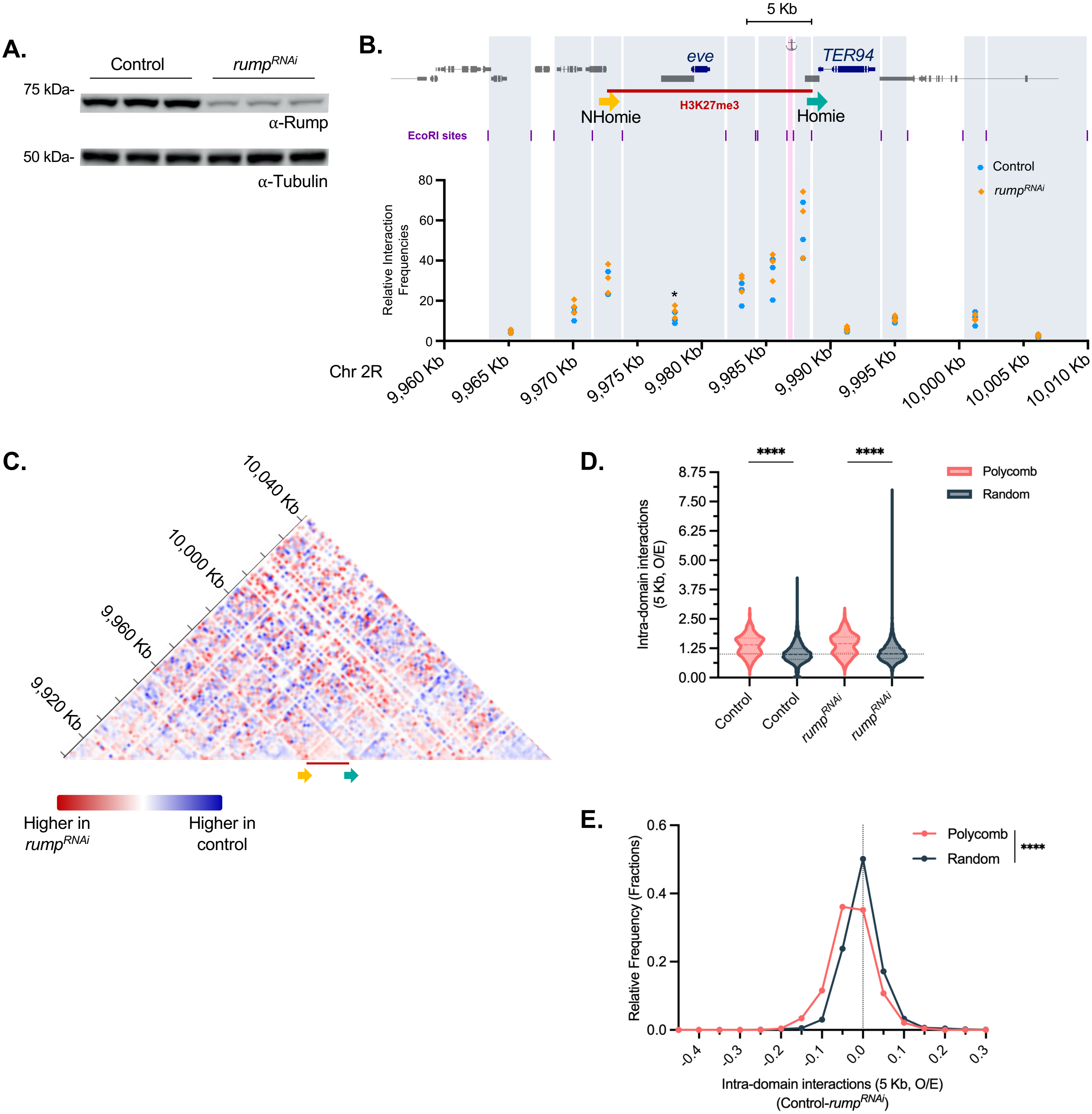

Additional Figure 12: Neither CTCF nor Cp190 alter *cis*-compaction at the *eve-TER94* locus

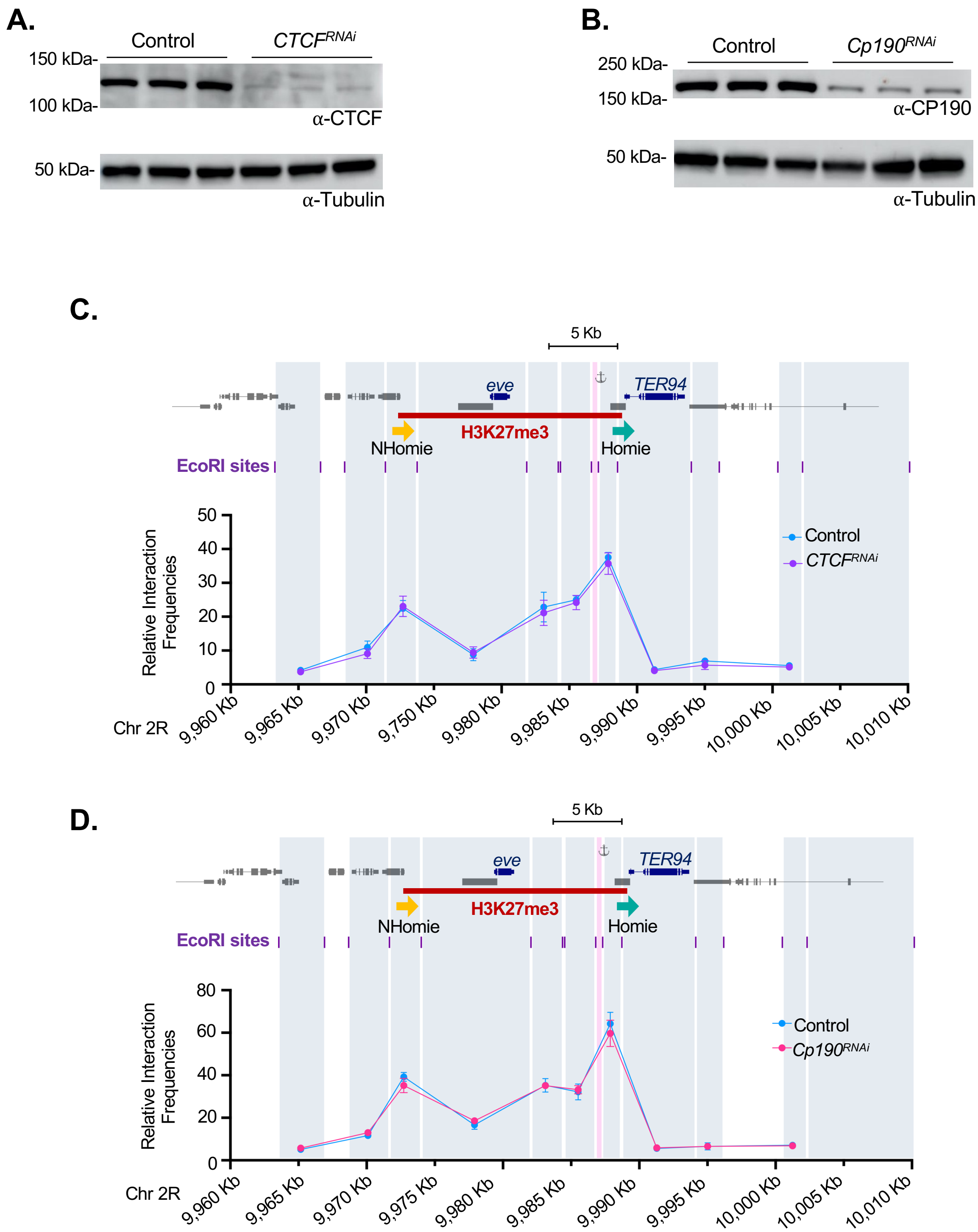

Additional Figure 13: Polycomb domain interactions are reduced, and Polycomb domain probe distances are increased after Rump depletion.

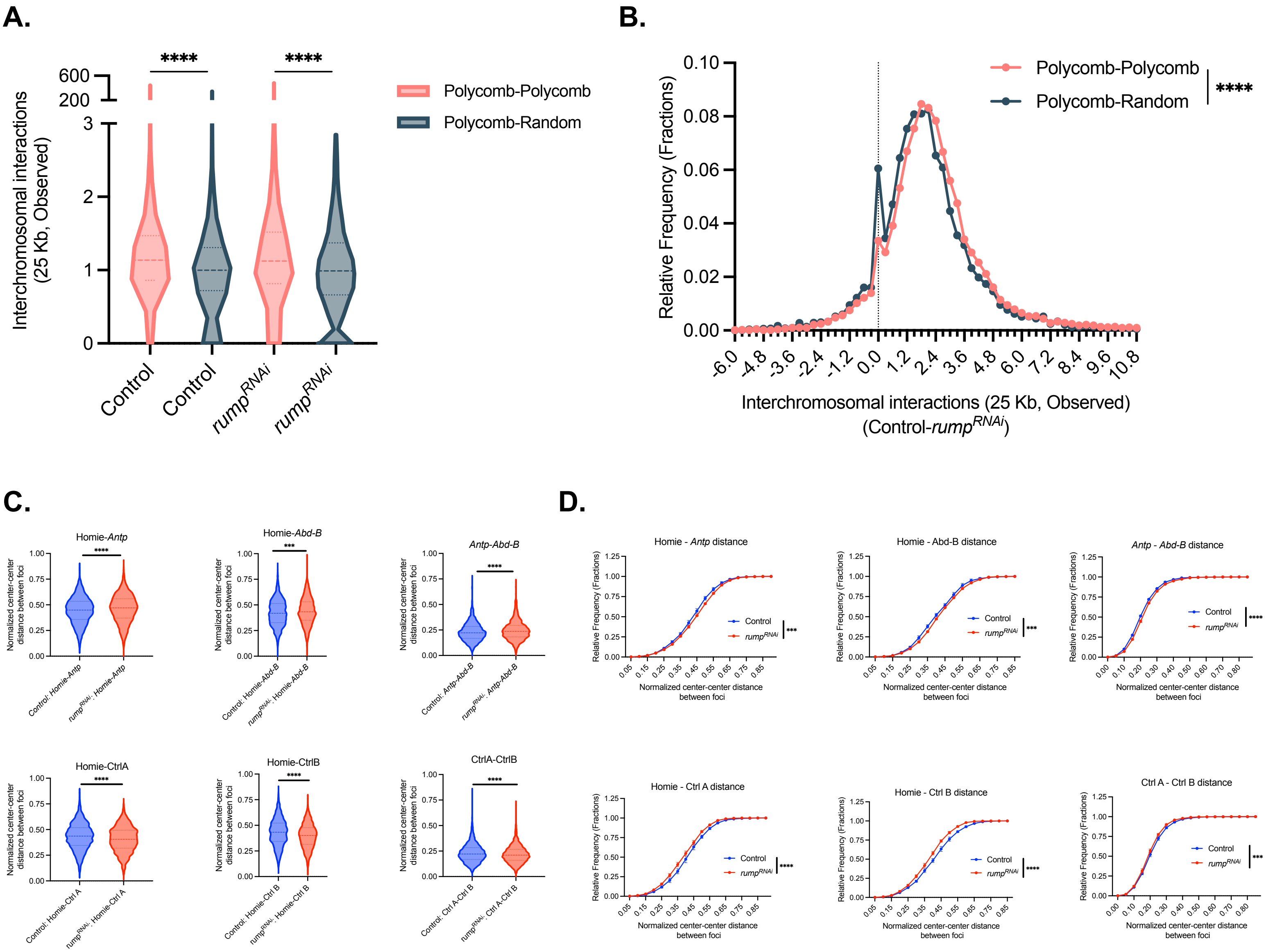

Additional Figure 14: Non-Polycomb domain probes are closer together after Rump depletion

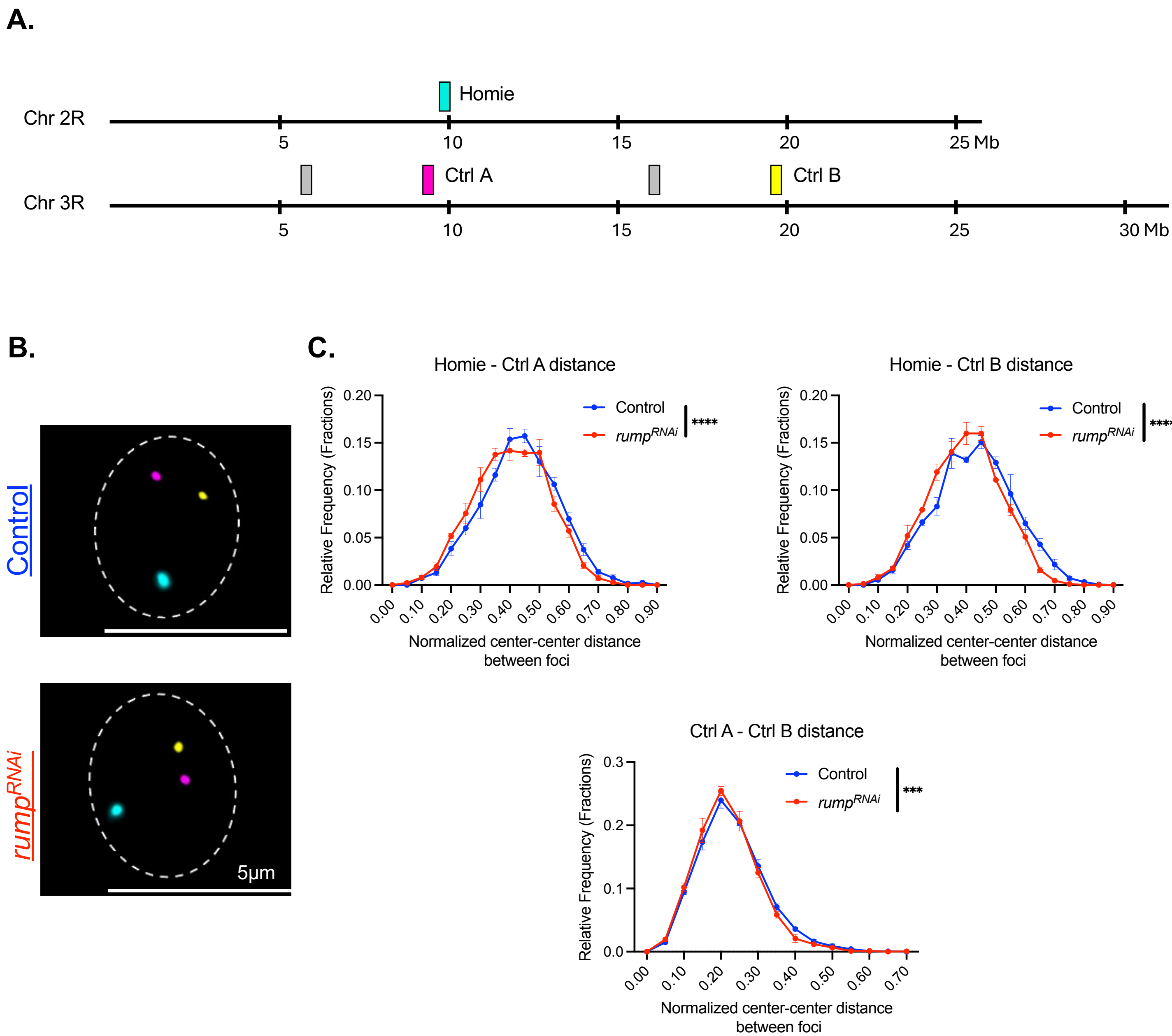

Additional Figure 15: CTCF and CP190 largely have no effect on inter-Polycomb/non-Polycomb probe distances.

A.

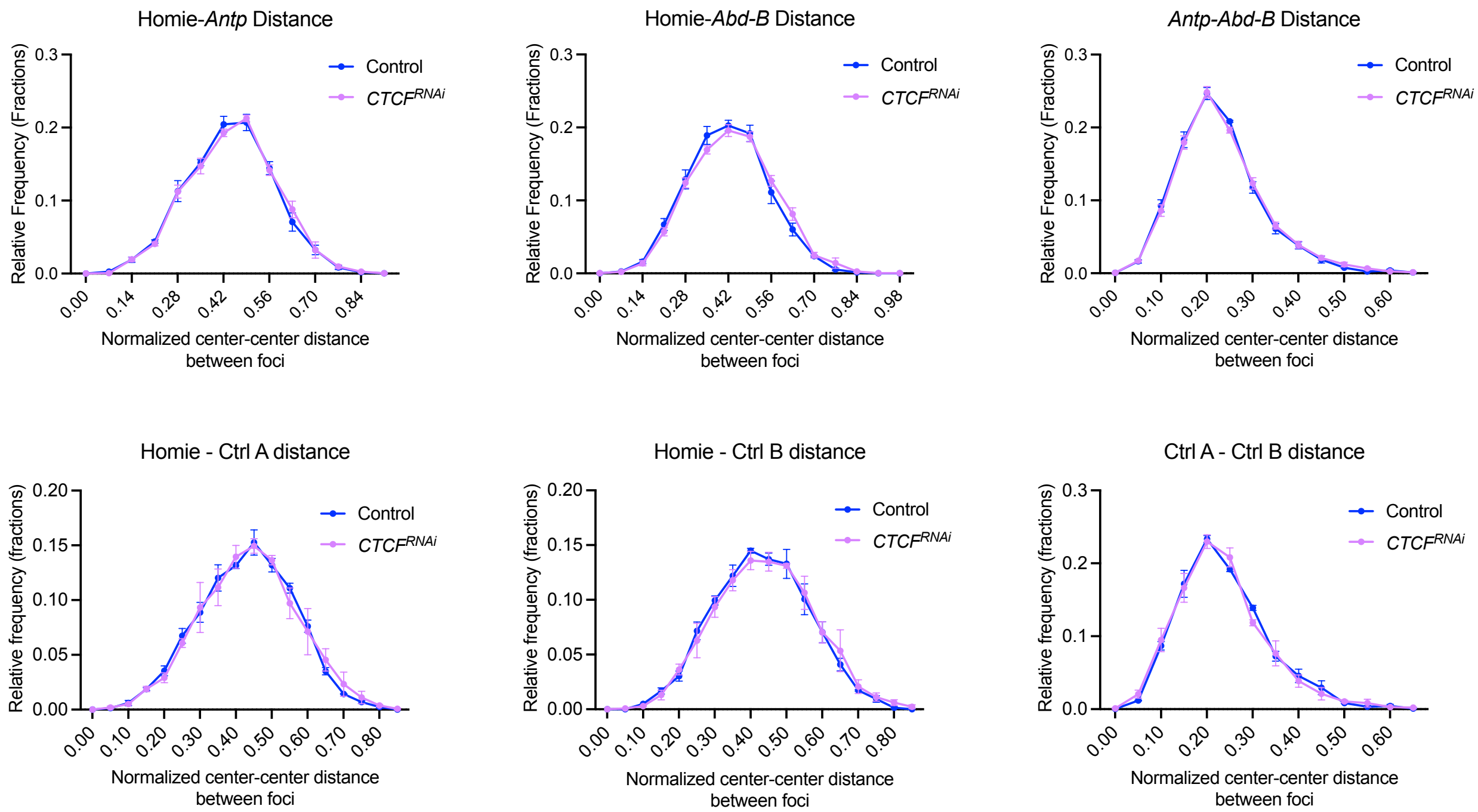

B.

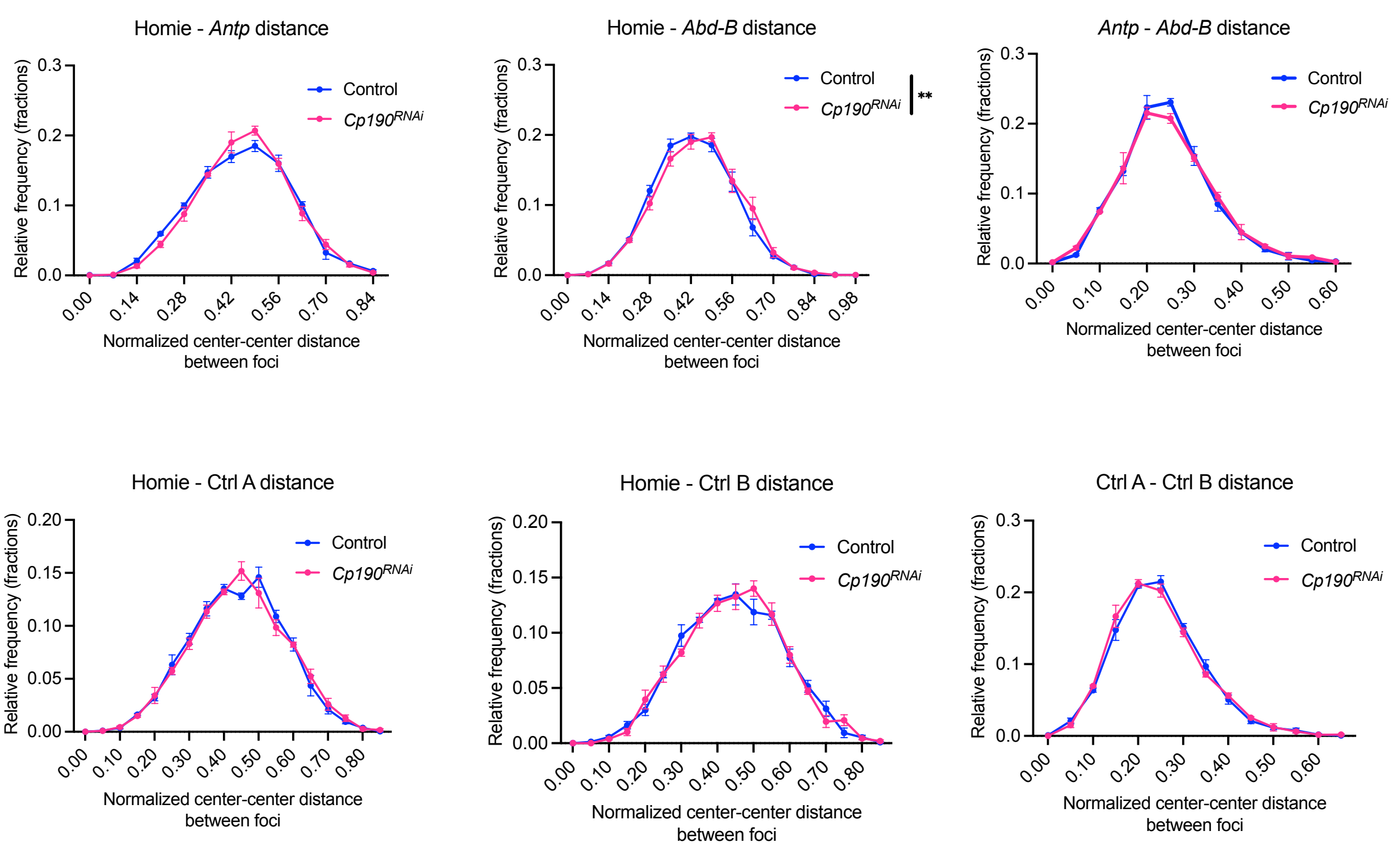

Supplement: Supplementary file 1 [file 13072_2026_694_MOESM1_ESM.pdf]
